# Nucleotide binding as an allosteric regulatory mechanism for *Akkermansia muciniphila* β-*N*-acetylhexosaminidase Am2136

**DOI:** 10.1080/19490976.2022.2143221

**Published:** 2022-11-17

**Authors:** Chang-Cheng Li, Huan Yi, Yan-Mei Wang, Xin-Yue Tang, Yi-Bo Zhu, Ying-Jie Song, Ning-Lin Zhao, Qin Huang, Xing-Yu Mou, Gui-Hua Luo, Tong-Gen Liu, Gang-Long Yang, Yu-Jiao Zeng, Li-Jie Wang, Hong Tang, Gang Fan, Rui Bao

**Affiliations:** a Division of Infectious Diseases, State Key Laboratory of Biotherapy and Center of Infectious Diseases, West China Hospital, Sichuan University, Chengdu, China; b State Key Laboratory of Southwestern Chinese Medicine Resources, College of Ethnic Medicine, Chengdu University of Traditional Chinese Medicine, Chengdu, China; c Institute of traditional Chinese medicine, Sichuan College of traditional Chinese Medicine (Sichuan Second Hospital of TCM), Chengdu, China; d School of Biotechnology, Jiangnan University, Chengdu, China

**Keywords:** *Akkermansia muciniphila*, glycosidase, mucin, protein structure

## Abstract

β-*N*-acetylhexosaminidases (EC3.2.1.52), which belong to the glycosyl hydrolase family GH20, are important enzymes for oligosaccharides modification. Numerous microbial β-*N*-acetylhexosaminidases have been investigated for applications in biology, biomedicine and biotechnology. *Akkermansia muciniphila* is an anaerobic intestinal commensal bacterium which possesses specific β-*N*-acetylhexosaminidases for gut mucosal layer colonization and mucin degradation. In this study, we assessed the in vitro mucin glycan cleavage activity of the *A. muciniphila* β-*N*-acetylhexosaminidase Am2136 and demonstrated its ability that hydrolyzing the β-linkages joining *N*-acetylglucosamine to a wide variety of aglycone residues, which indicated that Am2136 may be a generalist β-*N*-acetylhexosaminidase. Structural and enzyme activity assay experiments allowed us to probe the essential function of the inter-domain interactions in β23-β33. Importantly, we revealed that the hydrolysis activity of Am2136 was enhanced by nucleotides. We further speculated that this activation mechanism might be associated with the conformational motions between domain III and IV. To our knowledge, this is the first report of nucleotide effector regulated β-*N*-acetylhexosaminidase, to reveal its novel biological functions. These findings contribute to understanding the distinct properties within the GH20 family and lay a certain foundation to develop controllable glycan hydrolyzing catalysts.

**Abbreviations**: OD600 - optical cell densities at 600 nm; LB - Luria–Bertani; IPTG - isopropyl β-D-1-thiogalactopyranoside; PMSF - phenylmethanesulfonyl fluoride; rmsd - root mean square deviation; GlcNAc - N-acetyl-β-D-glucosamine; GalNAc - N-acetyl-β-D-galactosamine; Gal - galactose

## Introduction

Oligosaccharide contains different sugar units, due to its wide variety of biological activities, it has potential commercial values in food, pharmaceutical and cosmetic industries.^1^ β-*N*-acetylhexosaminidase is one of the most abundant glycosidases and is specific for the hydrolysis of both β-GlcNAc and β-*N*-acetylgalactosamine (β-GalNAc) units from the non-reducing end of glycan chains.^2^ Because of the ability to degrade a wide variety of substrates, and the wide resources including bacteria, fungi and arthropods,^3,4^ various therapeutic and biotechnological applications have been proposed for β-*N*-acetylhexosaminidase, which include conversion of industrial-scale production of distinct functional carbohydrates and glycan derivatives.^5^

Christoph Mayer and Vladimír Křen *et al*. have proposed a classical catalysis mechanism of β-*N*-acetylhexosaminidase: the “double-displacement mechanism” requires a nucleophile amino acid Asp/Glu to attack the substrate to form an enzyme-substrate complex intermediate, then a water would subsequently attack the intermediate and transfers a proton to the acid/base.^3^ The “substrate-assisted mechanism” is also a kind of “double displacement mechanism”, while they only differ from their intermediates. The HexNAc from the substrate acts as a nucleophile to attack the Asp/Glu and forms an oxazolinium ion intermediate, catalytic Asp/Glu residue provides protons to activate the water and finally generate the reaction product.^6^ Based on the distinct mechanisms, structures and functions, the known exo-β-*N*-acetylhexosaminidases have been grouped into several families including GH3, GH5, GH20, GH84, GH109 and GH116, although the oxidative mechanism of GH109 is still missing.^7^ The GH20 family has the largest number of β-*N*-acetylhexosaminidases, and they generally utilize the substrate-assisted mechanism.^8^ In GH20, the enzymes from eukaryotes are dimer and each monomer has two subunits,^9^ while that from prokaryotes possess diverse structures. Increasing structural studies keep expanding our understanding of correlations between the function and structure in glycosidase. The enzyme SpHex from *Streptomyces plicatus* only has two conserved subunits,^10^ but the enzyme GcnA from *Streptococcus gordonii* ^11^ and SmGH20A from *Serratia marcescens* ^12^ have three and four subunits, respectively. The different structures confer the enzymes various functions, some of them even participate in the synthesis of oligosaccharides.^13^

GH20 enzyme with β-*N*-acetylhexosaminidase activity have a wide range of cellular functions across different organisms, ranging from cell growth control, to host–pathogen interactions. For instance, gut microbes have to release specific mucin-degrading enzymes such as β-*N*-acetylhexosaminidases to regulate intestinal microecology.^14^ *Akkermansia muciniphila* is a famous mucin-degrading intestinal commensal bacterium, and extensive studies have revealed that *A. muciniphila* has many clinical benefits.^15,16^ The outstanding mucin-degrading capability of *A. muciniphila* is mainly caused by its wide variety and amounts of glycosidases.^17^ There are more than 3% genes encoding β-*N*-acetylhexosaminidases in *A. muciniphila* genome,^18^ and although a few of them have been reported,^19–21^ the detail of their distinct structural and biochemical properties still needs further studies. Recently, a novel GH20 glycosidase Am2136 characterized by its additional domain has been reported.^20^ However, the detail of the function and mechanism is not yet clear. In this article, we gained the unique crystal structure of Am2136, and revealed the ability of Am2136 to act on a wide range of different oligosaccharides. Importantly, we found that the catalytic activity of Am2136 can be regulated by nucleotides and made a certain explanation for the mechanism based on the structure and biochemical experiment data. In general, our results indicated the potential role of Am2136 in the metabolism of sugar nucleotide and laid a foundation for further development of novel GlcNAcase with fine-tuned activities which can be controlled by varying the concentration of allosteric modulators.

## Materials and methods

### Bacterial growth conditions and gene cloning

*A. muciniphila* (ATCC BAA-835) was cultured under anaerobic conditions at 37°C in Brain Heart Infusion (BHI) media supplemented with 0.05% (w/v) hog gastric mucin type III (Sigma Aldrich). Growth was measured by spectrophotometer as optical density at 600 nm (OD600) (NanoDrop 2000, Thermo Fisher).

The coding region (residue Q20-L756) of Am2136 without signal peptide was based on UniProt ^22^ and obtained through PCR on Gene Amplification Machine (Gene Touch, BIOER, HangZhou, China). The coding gene (Amuc_2136) was cloned into pET-22b with a 6XHis-tag labeled in the N terminal by using ClonExpress II One Step Cloning Kit (Vazyme) to generate pET22b-His-Amuc_2136. Based on this template, the mutants were constructed by using the QuickChange (High Fidelity Master Mix, MCLAB) PCR-based method. All the recombinant plasmids were further verified by DNA sequencing.

### Protein expression and purification

The recombinant plasmid was transformed into E. *coli* strain BL21 (DE3) for expression. A single colony was used to inoculate 50 ml of Luria-Bertani (LB) medium containing 30 μg/ml Ampicillin, and shook at 37°C overnight. Then, 5 ml portions were used to inoculate four 2 L flasks, each of which contained 1 L of the same medium. The cultures were allowed to grow with moderate shaking (220 rpm) at 37°C to an optical density (OD) at 600 nm of 0.8 and then cooled down to 16°C. The expression was induced by adding isopropyl β-D-thiogalactopyranoside (IPTG) to 0.05 mM and the culture was shaking for an additional 12–14 h at 16°C.

As for the expression of selenium-labeled Am2136, the recombinant plasmid was transformed into E. *coli* strain B834 (DE3). A single colony was still cultivated in the LB medium and shook at 37°C overnight. The LB medium would be removed by centrifugation at 4000 rpm for 15 min at 4°C, and then the bacteria were cultured in the M9 medium supplemented with various amino acids and vitamins at 37°C until its

OD_600_ reaching 0.8. Finally, 0.05 mM IPTG was added and induced for 16 h at 16°C.

All the cells were harvested by centrifugation at 4000 rpm for 15 min at 4°C and suspended with lysis buffer containing 25 mM Tris-HCl (pH 8.0), 150 mM NaCl, 5% glycerol and 1 mM phenylmethanesulfonyl fluoride (PMSF) and then lysed by sonication. The lysate was cleared by centrifugation at 17,000 g for 30 min at 4°C, and the supernatant was incubated with the Ni-NTA resin (Sigma Aldrich) for 60 min at 4°C. The Ni-NTA column was washed with lysis buffer and then eluted with elution buffer (25 mM Tris-HCl pH 8.0, 150 mM NaCl, 5% glycerol, 350 mM imidazole). The eluted protein solution was concentrated to 1 ml by using Centricon filter (50 kDa cutoff; Millipore) and exchanged into gel filtration buffer (25 mM Tris-HCl pH 8.0, 150 mM NaCl, 5% glycerol) using the column Superdex 200 Increase 10/300 GL (GE Healthcare) at 0.5 ml/min at 4°C. The protein was snap frozen in liquid nitrogen and stored at −80°C. The protein used for MS analysis was purified with volatile buffer (150 mM NH_4_Ac, pH 8.0), and the other process remains unchanged.

### Crystallization, structure determination and refinement

The protein samples were concentrated to 6.5 mg/ml for crystallization experiments. Initial crystallization experiments were carried out in 96-well Mosquito (TTP LabTech Ltd.) plates with commercially available crystallization screens from Hampton Research and Rigaku (Index HT, Crystal Screen HT, WIZARD HT, Xtal Quest HT, Salt RX HT, PEG RX HT). Crystallization was performed at 293 K by the hanging-drop vapor diffusion method, the 200 nL mixing drop containing protein solution and reservoir buffer in 1:1 ratio. The final optimized crystals were obtained from 0.2 M Li_2_SO_4_, 25% w/v PEG3350, 0.1 M HEPES, pH 7.5. The crystals were transferred to the reservoir solution plus 20% Glycerol and flash-cooled in liquid nitrogen. Diffraction data were collected with a CCD camera on BL-18 U stations in Shanghai Synchrotron Radiation Facility (Shanghai, China). Data processing and scaling were carried out using the HKL2000 software package.^23^

The data of selenium-labeled Am2136 was processed to a resolution limit of 2.9 Å in space group P12(1)1, with unit-cell parameters of a = 96.20 Å, b = 119.51 Å and c = 161.93 Å. The diffraction data were processed with HKL2000, and the phases were calculated using AutoSol.^24^ The structure refinement was performed using COOT ^25^ and PHENIX,^26^ and the structural representations were made through PyMOL.^27^

### Molecular docking

The solved Am2136 apo structure was first processed with the use of PyMOL, and docking was performed with AutoDock 4.2.6.^28^ Additional molecules were deleted and the protonation state of the structure was adjusted for neutral pH. The grid box of 40 × 40 × 40 points was used with a spacing 0.375 Å, and the grid box center was put on *x* = −20.101, *y* = 18.175, and *z* = −58.633. Gasteiger charges were assigned to protein and ligand molecules, and the molecule center is about 43.151, −13.360, 79.262. Exhaustiveness was set on 200 and a computer with eight processors was utilized for the computation. Hundred poses were generated for GDP and preparation of the image representing the best pose was done with PyMOL. An additional blind docking was also performed with the use of the Swissdock web server (http://swissdock.vital-it.ch/).^29^

### Enzyme assays of Am2136 wild-type and mutants

The activity was determined by measuring the change of 405 nm absorption of the liberated 4-nitrophenolate, and there are 51 time points measured within 5 min on Cytation3 (BioTek). The measurement was conducted under 37°C using *p*-nitrophenyl (*p*NP)-β-GlcNAc (Sigma) as a substrate. The 50 μL reaction mixture contains 0.125–4 mM substrate, 25 mM Tris pH 8.0, 150 mM NaCl, and the reaction was started by adding proteins to 2 μM final concentration. In order to study the influence of the nucleotides on the enzymatic activities, 1 mM NDP or NTP were introduced to the reaction system. All experiments were repeated three times.

### The metal ion effect on enzyme hydrolysis activity

The purified protein was added with the final concentration of 5 mM EDTA and incubated on ice for 20 min. Then the protein was dialyzed with the buffer solution (2 mM EDTA, 25 mM Tris, 150 mM NaCl, 5% Glycerol, pH 8.0) at 4°C for 4 h at a volume ratio of 1:350. After that, the protein was dialyzed with the EDTA-free buffer for 4 h and repeated twice.

The dialyzed protein was incubated with other metal ions on ice for 5 min. The 50 μL reaction mixture contains 2 μM protein, 0.125–4 mM *p*-nitrophenyl (*p*NP)-β-GlcNAc, 2 mM metal ions (MgCl_2_, CaCl_2_, CuCl_2_, ZnCl_2_, CoCl_2_), 25 mM Tris pH 8.0 and 150 mM NaCl. There are 51 time points measured within 5 min on Cytation3, and the activity was determined by measuring the change of 405 nm absorption of the liberated 4-nitrophenolate.

### The nucleotide binding affinity

The nucleotide binding ability was determined on Fluorescence Spectrometer Duetta (HORIBA) by measuring the change of peak intensity. The fluorescence was excited at 286 nm and emitted at 336 nm. And the measurement was processed under 400 V voltage with 2.5 nm excitation slit and 5 nm emission slit. The 120 μL reaction mixture contains 0–550 μM nucleotide, 5 μM proteins, 25 mM Tris pH 8.0 and 150 mM NaCl. The sample was incubated at 4°C for 5 min before testing.

### The capability of mucin hydrolysis

The activity of mucin hydrolysis of recombinant Am2136 was first probed with thin-layer chromatography (TLC). The 100 μL reaction mixture contains 10 mg/ml hog gastric mucin (type III, Sigma), 100 μM proteins, 150 mM NH_4_Ac pH 8.0. After reacting at 37°C for about 14 h, the samples were centrifuged at 12,000 rpm for 3 min at room temperature. The supernatant was spotted on a silica gel plate and allowed to air dry. The silica gel plate was then placed in a chamber containing a running buffer of 1-butanol, absolute ethanol and ddH_2_O at a ratio of 5:3:2. The silica gel plate was dried and then visualized using a buffer containing 2% acetone diphenylamine, 2% acetone aniline and 85% phosphoric acid at a ratio of 5:5:1 with heating at 110°C for 15 min.

The samples were heated at 100°C for 5 min and centrifuged at 15,000 rpm for 30 min before further separated by high-performance liquid chromatography-evaporative light scattering detector (HPLC-ELSD). HPLC (UltiMate 3000, Thermo Fisher) was carried out on WELCH Ultimate® XB-NH2 column (4.6 × 250 mm, 5 μm) under 30°C. The mobile phase was 70% acetonitrile (ACN) and eluted at a flow rate of 1 ml/min, and the injection volume was 2 μL. The effluent was monitored by ELSD detector (Alltech 2000ES) with 90°C drift tube temperature. The nitrogen flow rate is 2 L/min, and the value of gain is 1.

The repeated samples were separated without detecting and then collected followed by the analysis of ultrafleXtreme MALDI-TOF/TOF MS (Bruker Daltonics). All spectra were obtained in reflectron mode with an acceleration voltage of 24.59 kV, a reflector voltage of 26.6 kV, and a pulsed ion extraction of 100 ns in the positive ion mode. TOF/TOF was used to measure the fragmented glycan ions. Precursor ions were accelerated to 7.38 kV and selected with a timed ion gate. Fragment ions generated by the laser-induced decomposition of the precursor were further accelerated to 18.98 kV in the LIFT cell. Finally, the data would be analyzed by FlexAnalysis 3.3, and the glycan structures were annotated by GlycoWorkbench 2 with a signal–noise ratio more than 6.

### The capability of various oligosaccharides hydrolysis

The 30 μL reaction mixture contains 1.2 mM oligosaccharides (Sigma), 4 μM proteins, 150 mM NH_4_Ac pH 8.0. The qualitative and quantitative analyses were performed by high-performance liquid chromatography-tandem triple quadrupole mass spectrometry (HPLC-QqQ-MS) (Agilent 1260 HPLC system and 6460 QqQ system, Agilent Technologies, USA). The separation was performed on a Chromplus C_18_ column (4.6 × 250 mm, 5 μm) at 25°C. The samples were isocratic eluted with 0.1% formic acid in water and acetonitrile (80:20, v/v) at a flow rate of 0.5 mL/min with an injection volume of 5 μL. The mass spectrometer was operated in the electron spray ionization mode with drying gas temperature of 300°C, N_2_ gas flow at 11 L/min, nebulizer pressure of 15 psi, and capillary voltage of 4000 V.

Qualitative analysis was carried out by MS2 full-scan mode, while quantitative analysis was performed by multiple reaction monitoring (MRM) in positive ionization mode. Amygdalin (IS) was used as internal standard, and the sample was prepared by adding 20 μL internal standard (116.96 μM) and 170 μL water to 10 μL reaction mixture. The ion transitions were chosen as m/z 222.0→203.9 for GlcNAc and m/z 458.1→296.0 for IS. The fragmentor voltage values of GlcNAc and IS were set to 70 and 130 V, respectively. The collision energies of GlcNAc and IS were set to 1 and 10 eV, respectively. The signal acquisition and peak integration were performed using the MassHunter Qualitative Analysis Software (Agilent, USA). The concentration of GlcNAc in each sample was determined by using the established calibration curve (y = 0.768x + 2.4873) made from the standard compound.

## Results and Discussion

### The mucin cleavage capacity of Am2136

The gastric mucin secreted by gut epithelial is a kind of high molecular-weight glycoprotein, and the main compositions of the glycan chain are *N*-acetyl-D-glucosamine (GlcNAc), *N*-acetyl-D-galactosamine (GalNAc), galactose (Gal), fucose and sialic acid.^30^ Various monosaccharides derived from cleaved mucin glycans are not only the energy source for gut microbes but also important substrates for functional short chain fatty acids (SCFA) synthesis.^15^ *A. muciniphila* has evolved out distinct glycosidases to consume the mucin glycans.^31^ As the major group of GH20 family glycosidase, β-*N*-acetylhexosaminidases are responsible for the efficient mucin degrading capability of *A. muciniphila* and show immense structural and functional diversity. Previous reports have indicated that β-*N*-acetylhexosaminidase Am2136 could act on both *p*NP-β-GlcNAc and *p*NP-β-GalNAc, but with more preference towards *p*NP-β-GlcNAc.^20^ In order to verify the mucin hydrolysis ability and glycosidic linkage specificity of the substrate, we incubated the hog gastric mucin with Am2136 under 37°C for 14 h, then analyzed the reactant with thin-layer chromatography (TLC). The standards of Mannose and Glucose were used to test the reaction system (Fig S1A). Compared with Fig S1B, a ladder of vertical migration in Fig S1C indicated that carbohydrates were generated from the Am2136 treated groups. This is inconsistent with the HPLC-ELSD analysis as the extra peaks (retention time 7.5–9 min) were only observed in Am2136 treated mucin (Fig S1D-F). As the extra peaks (retention time 7.5–9 min) were composed of various oligosaccharides (Fig S1G), we deduced that Am2136 is active on the mucins.

**Figure 1.**
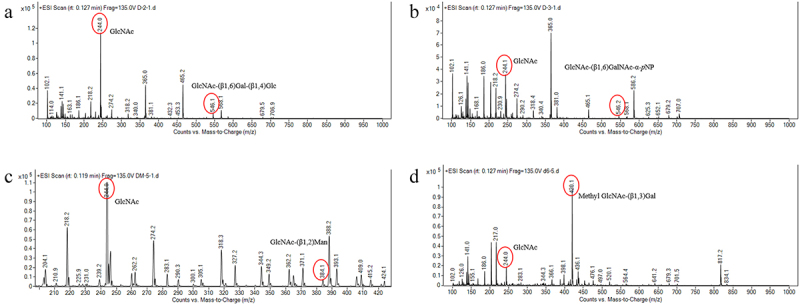
The MS spectrum of various oligosaccharides treated with Am2136. **a-c**. The MS spectra [M + Na]^+^ peaks of released GlcNAc and [M + H]^+^ peak of remanent GlcNAc-(β1,6)Gal-(β1,4)Glc, GlcNAc-(β1,6)GalNAc-α-*p*NP and GlcNAc-(β1,2)Man, respectively. **d**. The MS spectra [M + Na]^+^ peaks of released GlcNAc and Methyl GlcNAc-(β1,3)Gal.

Combining from the previous studies on the glycosyl composition of mucin,^30^ we incubated Am2136 with various oligosaccharides including GlcNAc-(β1,6)Gal-(β1,4)Glc, GlcNAc-(β1,6)GalNAc-α-*p*NP, GlcNAc-(β1,2)Man and Methyl GlcNAc-(β1,3)Gal and analyzed the products with QqQ-MS. The structure information of these carbohydrates and Amygdalin (IS) is present in Fig S2A-F. Fig S3 showed the MS spectrum of GlcNAc and oligosaccharide substrate before reaction. After treated with Am2136 for 1 h under 37°C, the extra peak of GlcNAc appeared in Figure 1a-d indicated that Am2136 could hydrolyze the glycosidic bond which is next to the 2-acetamido groups in all the tested substrates which containing the non-reducing β-linkages joining *N*-acetylglucosamine. Generally, substrate with D-gluco-structures is preferred for most β-*N*-acetylhexosaminidases, the GlcNAcase activity is 1.5 to 4.0 times higher than GalNAcase.^32^ The high GlcNAcase/GalNAcase ratio (~100 times) found in Am2136 ^20^ represents one of the special glycosidases involved in mucin degradation.

### Nucleotides have positive effect on Am2136 hydrolysis ability

**Figure 2.**
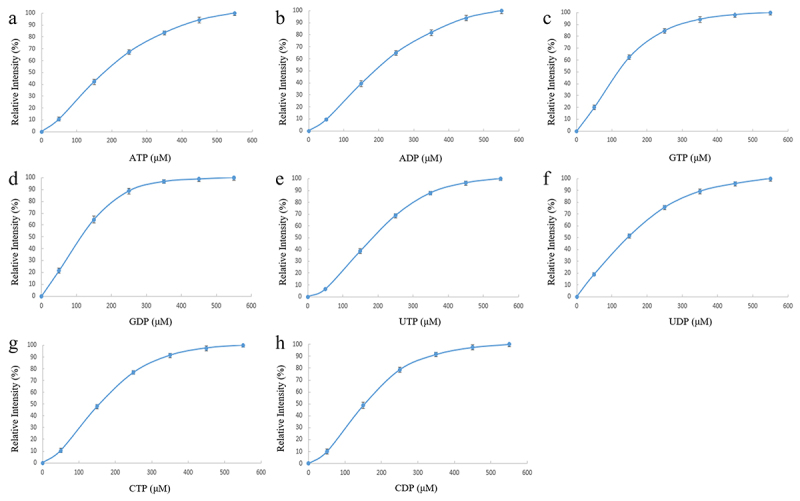
A-H. The binding curve of different nucleotides (ATP, ADP, GTP, GDP, UTP, UDP, CTP, CDP) binding to the wild type Am2136. The relative intensity indicates the normalized fluorescence value of protein affected by the nucleotides under various concentrations.

Among the known GH20 family members, Am2136 is unique for its possession of additional N-terminal domain (D21-G107) and C-terminal galectin-like domain (D601-L756).^20^ The galectin-like domain usually acts as a sugar moiety-recognition module.^33^ Thus, we evaluated the interactions of Am2136 with laboratory available carbohydrates and nucleotide molecules by fluorometry using the intrinsic fluorescent properties of the protein. Intriguingly, only nucleotide exhibited significant binding to Am2136 (Figure 2). The calculated *K_D_* values indicated that the nucleotide-binding was specific in which the base type and phosphate group might contribute to selectivity (Table 1).

**Table 1.** The parameters of the binding between Am2136 and various nucleotides.

Protein	Nucleotide	*K_D_* (μM)
Am2136 WT	ATP	245.13 ± 5.99
	ADP	254.63 ± 4.98
	GTP	122.98 ± 3.76
	GDP	116.07 ± 6.46
	UTP	198.87 ± 3.66
	UDP	178.74 ± 7.71
	CTP	167.42 ± 5.91
	CDP	157.16 ± 3.89

**Figure 3.**
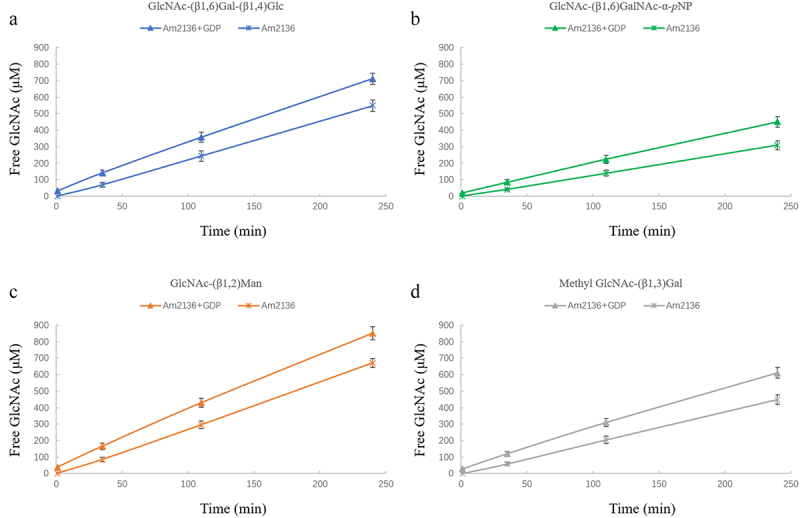
The quantitative analysis of effect of GDP on the Am2136 catalytic activity derived by HPLC-QqQ-MS. a-d. The amount of released GlcNAc from various substrates at different reaction time (1, 35, 110, 240 min). The oligosaccharides incubated with Am2136 are indicated with asterisk, and those treated with Am2136 and GDP are marked with triangle. The substrate GlcNAc-(β1,6)Gal-(β1,4)Glc, GlcNAc-(β1,6)GalNAc-α-*p*NP, GlcNAc-(β1,2)Man and Methyl GlcNAc-(β1,3)Gal is colored in *blue, green, Orange* and *gray*, respectively.

Among the tested nucleotides, GDP showed the relatively highest binding affinity (*K_D_* = 116 μM) and thus was firstly selected for clarifying the effects of nucleotide-binding on Am2136 function. The enzymatic activity assay was performed by following the previously described protocol,^20^ substrate *p*NP-β-GlcNAc was used for measuring the released *p*-nitrophenol after Am2136 cleavage. Various concentrations of GDP (0–2.65 mM) were added into the reaction mixture and the results showed a concentration-dependent enhancement of Am2136 hydrolysis activity (Fig S4A), implicating a positive regulatory role of GDP. Then, 1 mM nucleotides were utilized for further analyzing their effects on kinetic parameters. Similarly, more or less increased Am2136 activities were observed (Table 2), and we could find that nucleotide-binding did not significantly affect the *V_max_* values but mainly reduced the *K_m_* values (from 1.46 mM to 0.7–0.85 mM). Furthermore, we used various oligosaccharides to confirm the hydrolysis capability of Am2136 and the modulation effects of nucleotide. The 1.2 mM substrate was incubated with 4 μM Am2136 under 37°C, and the product GlcNAc released at different reaction times was quantitatively analyzed by QqQ-MS. Amygdalin (Fig S2F) was used as internal standard (IS) to quantitatively measure the amount of free GlcNAc in the reaction mixture (Fig S4B). As shown in Figure 3a-d, after incubated with Am2136 for 240 min, there were 549 μM, 308 μM, 671 μM and 448 μM GlcNAc respectively, indicating the possible substrate preference order was: GlcNAc-(β1,2)Man > GlcNAc-(β1,6)Gal-(β1,4)Glc > Methyl GlcNAc-(β1,3)Gal > GlcNAc-(β1,6)GalNAc-α-*p*NP. In addition, when 1 mM GDP was added in the reaction mixture, the amount of free GlcNAc were increased to 712.55 μM, 451.25 μM, 851.33 μM and 610.53 μM respectively, without changing the substrate preference of Am2136 (Figure 3a-d). Therefore, the increase in Am2136-substrate affinity by the allosteric nucleotide binding may be a common mechanism for different substrates.

**Table 2.** The enzyme activity assays of Am2136 regulated by various nucleotides.

Protein	Nucleotides (1 mM)	*V_max_* (μmol min^−1^)	*K_m_* (mM)	*k_cat_*/*K_m_* (mM^−1^ s^−1^)
Am2136 WT	/	455 ± 7	1.46 ± 0.05	156 ± 4
	ATP	442 ± 9	0.85 ± 0.05	260 ± 10
	ADP	422 ± 6	0.86 ± 0.06	245 ± 12
	GTP	449 ± 7	0.82 ± 0.04	274 ± 9
	GDP	442 ± 7	0.70 ± 0.03	316 ± 8
	UTP	439 ± 7	0.84 ± 0.04	261 ± 8
	UDP	429 ± 9	0.91 ± 0.07	236 ± 13
	CTP	436 ± 7	0.84 ± 0.04	260 ± 8
	CDP	435 ± 6	0.81 ± 0.04	269 ± 10

Of the limited reports available on allosteric activator of glycosyl hydrolase, only anion effects on mammalian α-Amylase have been well documented, in which the chloride increases the *k_cat_* value but does not change the *K_m_*.^34^ While allosteric-binding compounds have been identified or designed for stabilizing human α-GalA (lysosomal enzyme α-galactosidase A).^35,36^ Inhibitory allosteric effects of T4 protein spackle on T4 phage gp5 lysozyme have been reported elsewhere.^37^ In this work, the maximal activation of Am2136 (up to twofold) was observed at 1 mM of GDP, indicating a high activation profile. Meanwhile, the presence of nucleotide is not obligatory for the catalytic reaction, suggesting that nucleotides act as non-essential activator. The unexpected finding of novel allosteric modulators of Am2136 provide further insight into the glycosidase mechanism.

### Structural characterization of Am2136 in apo form

**Figure 4.**
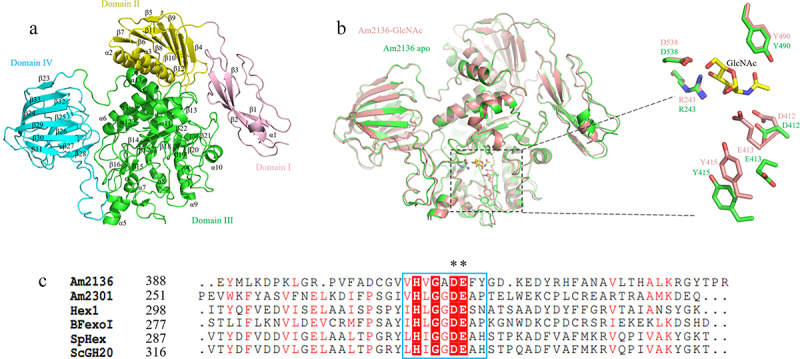
The detail of the Am2136 overall structure apo form. a. The Am2136 monomer structure is shown in cartoon, and the four subunits (domain I-domain IV) are colored in *pink, yellow, green* and *cyan*, respectively. **b**. The structural comparison of Am2136 apo form (*green*) and Am2136-GlcNAc complex (*cherry*). The crucial residues are shown in sticks, and GlcNAc is colored in *yellow*. **c**. The amino acid sequence alignments of essential residues of different β-*N*-acetylhexosaminidases in GH20. The crucial region is marked with *blue* box, the completely conserved residues are colored in *white* and shade in *red*, and the conserved residues are colored in *red*. The core residues D412 and E413 are indicated with asterisk.

The crystal structure of Am2136 (without signal peptide residues 1–19) in apo form was solved to 2.9 Å (Table 3). As Figure 4a shown, the catalytic domain III adopts a typical (β/α)8-barrel (TIM-barrel) fold which is structurally conserved in GH18, 20 and 85 GlcNAcases.^10,38,39^ Our refined Am2136 structure broadly resembles the previously reported Am2136-GlcNAc complex structure (PDB code:6JQF), with Cα atom root-mean square distance (rmsd) of 0.835 Å, demonstrating rigid structural integrity. However, by overlaying the domain III from these structures, we may find obvious structural variations at the substrate-binding sites. Most crucial residues involved in substrate GlcNAc moiety recognition could be structurally aligned well except residues D412, E413 and Y415 (Figure 4b). In contrast to the GlcNAc-bound structure (PDB code 6JQF), the side-chains of these catalytic residues adopt different orientations and protrude away from the reaction center in our structure. It has been widely recognized that GH20 enzymes catalyze reactions through substrate-assisted mechanism.^8^ From Figure 4c we can see that the region V406-Y415 is highly conserved among GH20 glycosidases from different species. Thus, Y415 is a crucial part of the hydrophobic pocket necessary for substrate binding, this interpretation is further supported by the obvious increased *K_m_* value (2.32 mM) of mutant Y415A (Table 4). The residue E413 provides a proton to the aglycone leaving group during the oxazolinium formation step, and then abstract one from the nucleophilic water during the oxazolinium hydrolysis, while D412 stabilizes the oxazolinium intermediate. Therefore, the ala-substitutions on these sites abolished the hydrolysis activity of Am2136

**Table 3.** The diffraction data collection and refinement statistics of Am2136.

	Am2136
**Data collection**	
Space group	P 1 2_1_ 1
**Cell dimensions**	
*a, b, c* (Å)	96.20, 119.51, 161.93
α, β, γ (°)	90.00, 103.40, 90.00
Wavelength	0.97930
Resolution (Å)	30–2.9 (3.02–2.90)
R_pim_	0.065 (0.244)
I/sigma CC_1/2_ No. of reflections	11.6 (3.0) 0.98 (0.890) 86,269 (8562)
Completeness (%)	99.2 (99.4)
Redundancy	13.7 (13.9)
**Refinement**	
Rwork Rfree	0.194 (0.279) 0.248 (0.345)
**No. of atoms**	
Protein	22,967
Ligand/ion	4
Water	202
Average B-factors	50.07
Protein	50.14
Ligand/ion	24.85
Water	42.52
**r.m.s.d**.	
Bond lengths (Å)	0.009
Bond angles (°)	1.36
**Ramachandran plot** Favored (%) Allowed (%) Outliers (%)	96.06 3.73 0.21

**Table 4.** The enzyme activity assays of Am2136 mutants which are related to subdomain interactions.

Protein	*V_max_* (μmol min^−1^)	*K_m_* (mM)	*k_cat_*/*K_m_* (mM^−1^ s^−1^)
WT	455 ± 7	1.46 ± 0.05	156 ± 4
D57A+E59A+Q60A	432 ± 11	1.41 ± 0.06	153 ± 3
D412A+E413A	NA	NA	/
T545A+G546A	264 ± 4	3.66 ± 0.69	36 ± 5
Δ D717-K727	144 ± 7	6.56 ± 0.41	11 ± 1
Y415A	399 ± 9	2.32 ± 0.08	86 ± 1

**Figure 5.**
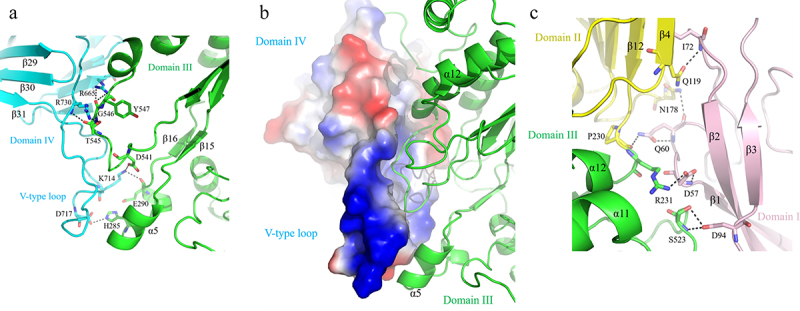
The structural analysis of interactions between subdomains. a. The interactions between domain IV (*cyan*) and domain III (*green*). The structure and key residues are shown in cartoon and sticks, respectively. The hydrogen bonds are indicated with *black* dash line. **b**. The electric potential distribution of domain IV. The *blue* and *red* region means polar and nonpolar residues, respectively. **c**. The interactions between domain I (*pink*) and domain II (*yellow*) and domain III (*green*).

The catalytic domain III was flanked by domain I, II and IV, in which the majority of interdomain contacts were established by the galectin-like domain IV (1099.1 Å^2^). This galectin-like module adopts a closing hand shape formed by two antiparallel β-sheet sandwiches (β23-β33). It forms several hydrogen bonds (R665-G546/Y547, R730-T545) and a charge–charge interaction (K714-D717) with domain III (Figure 5a), by which the extended V-type loop (V707-T732) can participate in the substrate-binding pocket formation (Figure 5b). Based on that, we generated T545A/G546A and ΔD717-K727 mutants in high homogeneity (Fig S5) to see their effects on kinetic properties. As expected, the *K_m_* value of mutant T545A+G546A was more than twice as much as that of wild type, and the apparent *V_max_* value was reduced to 264 μmol min^−1^ (Table 4). Similar but more attenuated effects were observed in ΔD717-K727 mutant. Meanwhile, various hydrogen bonds are also formed by residues from domain I, II and III (Figure 5c), among which residue D57 has been proposed to be part of the functional metal-binding region (D57, E59, Y232, Q233 and S523).^29^ However, in contrast with the mutations that disrupting domain III–IV interactions, the triple mutant D57A+E59A+Q60A had no obvious effects on the catalytic efficiency of Am2136 (Table 4). Hence, these results demonstrate that the specific interactions between domain III and IV are an important integral part of regulating the hydrolysis activity.

Up to now, the structures of over 26 GH20 family members have been solved. The conserved catalytic domain is commonly accompanied by several domains associated with diverse functions. Although *A. actinomycetemcomitans* dispersin B contains only a single GH20 domain,^40^ the minimal functional unit of most GH20 enzymes requires a non-catalytic domain named GH20b. GH20b has accessory but necessary roles in ensuring stable expression.^41^ Am2136 consists of four domains, in which the central II and III domains correspond to GH20b and GH20 domains separately. The N-terminal immunoglobulin-like domain I is a distinct module and is proposed to be essential for Am2136 stability, because the 21–107 truncated version is not stable enough.^20^ This seems to indicate that this unique domain I serves a similar function as GH20b in promoting protein folding. The domain IV bears structural resemblance to certain galectin,^42^ its potent substrate-binding property was taken into account, but incubation of the protein with carbohydrates did not result in any significant binding effects (results not shown). Nevertheless, whether or not its β sheet sandwich architecture could serve as a carbohydrate-binding module still needs further analysis. More specifically, structural analysis indicates that domain IV consists of an extended solvent-exposed loop to cover the side of catalytic domain. We show that disrupting the noncovalent association of domain IV with the catalytic domain significantly reduces the activity of Am2136 in kinetic analyses, and this effect could be attributed to the close spatial and functional association between the extended loop of domain IV and the edge of the substrate-binding pocket.

### Structural basis for the nucleotide recognition of Am2136

**Figure 6.**
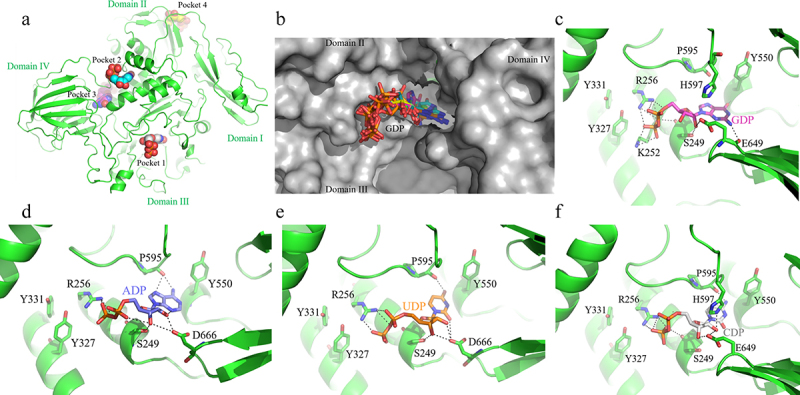
The structure model of docked Am2136-GDP complex. a. The different pockets in the overall structure of docked Am2136-GDP complex. The GDP in pocket 1–4 is shown in spheres and colored in *white, cyan, blue* and *yellow*, respectively. **b**. The superposition of top 10 results of docked GDP in pocket 3. The protein and GDP are shown in surface and sticks, respectively. **c-f**. The details of interactions between Am2136 and GDP (*purple*), ADP (*blue*), UDP (*Orange*) and CDP (*gray*), respectively. The nucleotides are shown in sticks, and the hydrogen bonds (*black*) are indicated with dashed line.

The phenomenological description of the linkage between nucleotide binding and hydrolysis activity of Am2136 encourages us to further explore its molecular mechanism. Although attempts have been made to crystallize nucleotide bound to Am2136, the complex structure has not been obtained. Therefore, we utilized the online prediction and docking to investigate the structural details. Firstly, the apo structure of Am2136 was submitted to binding site prediction server PrankWeb.^43^ Four possible ligand-binding pockets were identified, ranked as pocket 1–4 (Table 5 and Figure 6a). Pocket 1 with the highest score and is actually the substrate-binding site. While pocket 4 located on the top of β4 and β12 has the lowest probability, hence we excluded this site from the following docking experiments.

**Table 5.** The online analysis results of potential GDP binding pocket based on PrankWeb.

Name	Rank	Score	Probability	Center_X	Center_Y	Center_Z
Pocket 1	1	25.77	0.81	−28.6373	−1.7536	−75.9780
Pocket 2	2	3.53	0.066	−32.5954	22.1748	−63.4113
Pocket 3	3	2.45	0.031	−20.1008	18.1748	−58.6329
Pocket 4	4	1.16	0.004	−55.5634	24.2987	−41.4850

* The score means the sequence evolutionary conservation value of residues.

* Points with high predicted ligand ability are clustered and ranked according to a ranking function based on cumulative score of the cluster.

**Figure 7.**
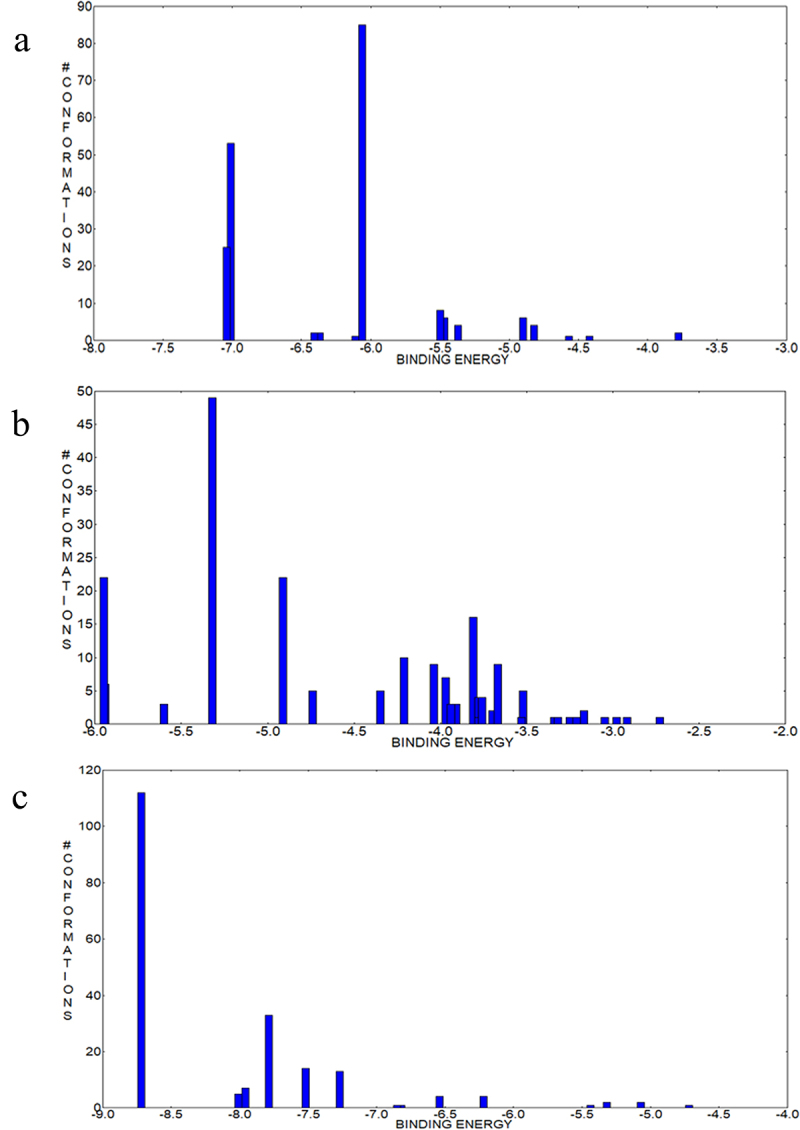
a-c. The cluster dendrogram of pocket 1, pocket 2, pocket 3 docking results, respectively. The horizontal axis represents the binding energy in different conformations, and the ordinate represents the number of conformations under the corresponding energy.

**Figure 8.**
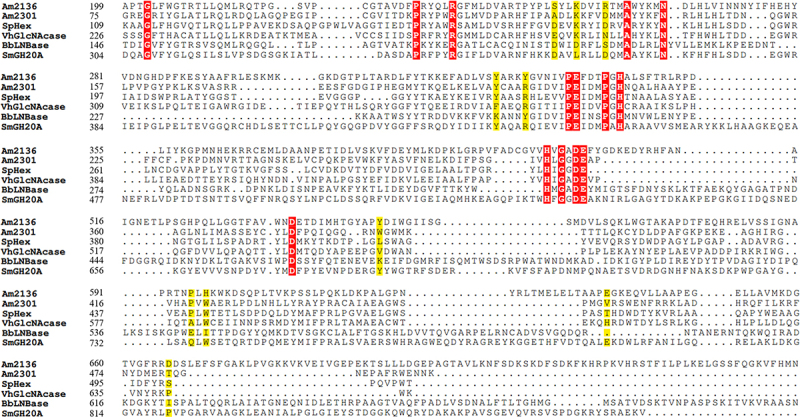
The amino acid sequence alignments of different.

The ligand GDP was docked with Am2136 apo structure using the AutoDock suite of tools. The center sites of predicted pocket 1–3 were used as grid centers and the docking parameters were set to 200 runs per protein-ligand complex. The clustering was performed at 2.0 Å r.m.s. to validate the convergence to the best pose, and the best pose was defined as the conformation possessing the least free binding energy. The clustering figure (Figure 7) shows that the least binding energy (−8.72 kcal/mol) and the best clustering (112 out of 200) are from the docking toward pocket 3 (Figure 6b). Analysis of the best docked complexes reveals that domain III and IV both participated in the GDP recognition. Five critical residues (S249, K252, R256, Y550, E649) that form direct interactions with GDP. The guanine moiety is fixed by forming hydrogen bonds with the main chain atoms of Y550 and E649 (Figure 6c). The side chains of E649 and S249 directly engage in ribose interactions, and basic residues R256/K252 may interact to form salt bridges with the phosphate group. Additionally, residues Y327, Y331, P595 and H597 that encompass the ligand-binding sites may offer ligand selectivity as well. As the nucleotides are structurally diverse, we also analyzed the binding mechanism of different nucleotides (ADP, UDP, CDP) via docking. According to the docking results with the lowest binding energy (Table 6), we can find that most interactions are conserved in different nucleotides binding, especially residues S249, R256 and P595 (Figure 6d-f). To explore whether these residues are conserved in other glycosidase, several GH20 enzymes with available structures (SpHex, VhGlcNAcase, BbLNBase, SmGH20A, and the PDB code is 1M01, 6K35, 4H04, 1QBB respectively) were structurally aligned with Am2301.^10,12,19,44–46^ It is obvious that the residues involved in binding pocket are not conserved (Figure 8), therefore the nucleotide regulation is a unique mechanism of Am2136.

**Table 6.** The docking results with lowest binding energy of Am2136 and nucleotides.\.

Ligand	Binding energy (kcal/mol)	Ligand efficiency	Inhibit constant (μM)	No. of H-bonds
ADP	−5.36	−0.2	117.88	7
GDP	−8.72	−0.31	407.67	11
UDP	−7.04	−0.28	6.91	8
CDP	−6.93	−0.28	8.27	10

The predicted nucleotide-binding site is similar to the ion-binding site observed in the previously published Am2136 structure (PDB code: 6JQF).^20^ We further demonstrated that, except Ca^2+^ and Mg^2+^, certain divalent metal ions including Cu^2+^, Zn^2+^ and Co^2+^ have significant inhibitory effects on Am2136 (Table 7). However, with or without Ca^2+^ or Mg^2+^, they did not exhibit obvious behavioral effect on GDP binding to Am2136 (Table 8), suggesting that the binding of nucleotides on this site is more specific than metal ions. The binding site and regulatory effects of allosteric effector are distinct from both substrate and product. Different allosteric features have been investigated toward nucleotide activators. Global re-arrangements including conformational changes both within the catalytic and regulatory modules are quintessential properties of CTP-regulated aspartate carbamoyltransferase.^47^ While for sterile alpha motif and HD domain-containing protein 1 (SAMHD1), additional nucleotides at allosteric sites would stabilize inter-monomer interactions thus support tetramer assembly.^48^ According to molecule docking calculations, the most probable nucleotide-binding site of Am2136 is found in the cleft between domain III and IV, located at more than 28.6 Å from the active site. This result is coincidentally in resonance with the observation that domain III-domain IV interaction is indispensable for the efficient hydrolysis activity, providing a possible mechanism to couple the allosteric effect with the interdomain associations.

**Table 7.** The effect of different metal ions on the Am2136 catalytic activity.

	Am2136	*V_max_* (μmol min^−1^)	*K_m_* (mM)	*k_cat_*/*K_m_* (mM^−1^ s^−1^)
	WT	455 ± 7	1.46 ± 0.05	156 ± 4
	WT dialyzed	475 ± 13	1.53 ± 0.08	155 ± 3
WT dialyzed +ions	+ Mg^2+^	435 ± 9	1.44 ± 0.06	151 ± 3
	+ Ca^2+^	447 ± 14	1.74 ± 0.09	128 ± 2
	+ Cu^2+^	NA	NA	/
	+ Zn^2+^	NA	NA	/
	+ Co^2+^	NA	NA	/

**Table 8.** The effect of calcium and magnesium on GDP binding.

	Am2136	Nucleotide	*K_D_* (μM)
	WT	GDP	116.07 ± 6.46
	WT dialyzed		113.65 ± 5.22
WT dialyzed +ions	+ Mg^2+^		118.23 ± 5.85
	+ Ca^2+^		116.53 ± 4.37

### Biochemical and mutational analysis of the putative GDP binding sites

In order to experimentally validate the silico studies on GDP-Am2136 interactions, single and double Ala substitutions were constructed in Y550, E649, R256 and P595/H597, and a mutation of a neighboring basic residue R592A was considered as a negative control. Y-to-F double swap was made on Y327/Y331 to define the roles of tyrosine in phosphate group recognition.

For enzymatic activity assay without GDP, the above mutants all retain the same kinetics profile as the wildtype while only Y550A and P595A/H597A exhibited slightly lower efficiencies (Table 9). In contrast, the positive allosteric GDP-modulation effects on substrate binding were largely impaired when mutations were introduced at those predicted GDP-anchoring residues (Table 10). In the presence of 1 mM GDP, the *K_m_* values of variant E649A, Y550A, Y327F/Y331F, and R256A were significantly less reduced than that of Am2136-WT. Comparing the catalytic efficiencies (*k_cat_*/*K_m_*) with and without GDP, the P595A/H597A showed a roughly two-fold increase, which is the same as the WT and control group R592A, indicating that those two sites are not the determinants for GDP binding.

**Table 9.** The enzyme activity assays of Am2136 wild type and mutants based on docking results.

Protein	*V_max_* (μmol min^−1^)	*K_m_* (mM)	*k_cat_*/*K_m_* (mM^−1^ s^−1^)
WT	455 ± 7	1.46 ± 0.05	156 ± 4
Y550A	430 ± 6	1.52 ± 0.04	143 ± 3
E649A	435 ± 15	1.45 ± 0.09	150 ± 4
R256A	447 ± 9	1.49 ± 0.06	150 ± 3
Y327F+Y331F	436 ± 8	1.42 ± 0.06	153 ± 3
R592A	445 ± 9	1.48 ± 0.06	150 ± 3
P595A+H597A	414 ± 9	1.58 ± 0.07	131 ± 3

**Table 10.** The GDP regulated enzyme activity assays of Am2136 mutants based on docking results.

Protein	Nucleotides (1 mM)	*V_max_* (μmol min^−1^)	*K_m_* (mM)	*k_cat_*/*K_m_* (mM^−1^ s^−1^)
WT	GDP	442 ± 7	0.70 ± 0.03	316 ± 9
Y550A		463 ± 7	1.24 ± 0.05	187 ± 5
E649A		453 ± 6	1.39 ± 0.04	163 ± 3
R256A		465 ± 15	1.08 ± 0.07	215 ± 7
Y327F+Y331F		473 ± 6	1.21 ± 0.04	195 ± 3
R592A		442 ± 5	0.75 ± 0.03	295 ± 8
P595A+H597A		447 ± 4	0.86 ± 0.03	260 ± 7

Intrinsic fluorescent spectroscopy measurements were carried out to further correlate the functional effects of these mutations on GDP-protein binding. As shown in Table 11, comparing with the WT and R592A, the GDP binding affinity of E649A was largely reduced (*K_D_* values increased from 116 μM to 236 μM). Thus, E649 plays a vital role for GDP recognition. Various degrees of weakened GDP binding were also observed in Y550A, Y327F/Y331F, and R256A. In agreement with the kinetic analysis, GDP binding is less sensitive to P595A/H597A mutation. Given the consistency of activity assay and GDP binding measurements, it is clear that the kinetics and regulatory properties of GDP are sensitive to residues Y550, E649, R256, Y327 and Y331, while

**Table 11.** The GDP binding parameters of Am2136 mutants based on docking results.

Protein	Nucleotide	*K_D_* (μM)
WT	GDP	116.07 ± 6.46
Y550A		192.51 ± 7.36
E649A		235.94 ± 24.49
R256A		157.89 ± 10.92
Y327F+Y331F		175.32 ± 13.75
R592A		120.05 ± 5.82
P595A+H597A		140.04 ± 4.26

P595 and H597 are partly involved in GDP recognition. Various degrees of weakened GDP binding were also observed in Y550A, Y327F/Y331F, and R256A. In agreement with the kinetic analysis, GDP binding is less sensitive to P595A/H597A mutation. Given the consistency of activity assay and GDP binding measurements, it is clear that the kinetics and regulatory properties of GDP are sensitive to residues Y550, E649, R256, Y327 and Y331, while P595 and H597 are partly involved in GDP recognition.

Structural data guided mutagenesis and kinetic studies are crucial for elucidating the synergy between regulatory and active sites. According to the structural and mutagenesis studies on SAMHD1,^48^ changes of residues that interacting with effector dGTP can significantly decrease the turnover rates, confirming the allosteric mechanism of activation through dGTP-promoted tetramerization and thereby inducing correct active site formation. From our results, any changes in nucleotide-binding pocket of Am2136 would result in decreased but not abolished allosteric effects, indicating a certain conformational tolerance in this binding site. Additionally, it is possible that the nucleotides binding provokes the conformational changes, and the allosteric activation resulted in an increase of the affinity between enzyme and substrate. Although the allosteric effect of nucleotide is not the “turn-on switch” for Am2136, the observed ligand specificity and “efficient activator” profile toward Am2136 suggest a possibility to identify or engineer novel and better performing β-*N*-acetylhexosaminidases.

## Conclusion

In this work, we purified the enzyme Am2136 and explored its potential broad carbohydrates cleavage capability. Intriguingly, we found that Am2136 had nucleotide-binding ability. Functional characterization demonstrated that the substrate binding affinity and hence enzymatic efficiency of Am2136 could be significantly improved by the addition of the di/triphosphate nucleotides. Therefore, Am2136 is the first reported glycosidase that exhibits allosteric activation in the presence of these nucleotide effectors. We further determined the crystal structure of Am2136 in apo form by the molecular replacement method and refined to 2.9 Å resolution. Specifically, inter-domain contacts between domain III and IV are crucial for its β-*N*-acetylhexosaminidase activity. Subsequently, molecular docking simulation and site-directed mutagenesis studies further revealed the critical amino acids involved in nucleoside effector recognition and regulation. The nucleotide-assisted activation property does not necessarily make Am2136 more efficient than other homologs (the highest enzymatic efficiency (*k_cat_*/*K_m_*) of GH20 β-*N*-acetylhexosaminidase listed in BRENDA database could reach up to 3428 mM^−1^ s^−1 49,50^), but makes it more flexible in regulation. Collectively, these results provided new insights into the mechanisms underlying mucin-degrading by microbial β-*N*-acetylhexosaminidase, and a useful basis for the elucidation of the possible biological significance of Am2136 in the functional process of *A. muciniphila*. Additionally, they also indicated that the further exploration on the novel adjustment of glycoside hydrolase will contribute to the β-*N*-acetylhexosaminidase utilization.

## Supplementary Material

Supplemental Material

## Data Availability

The data that support the findings of this study are openly available at https://data.4tu.nl/account/home, and the DOI number is 10.4121/19,564,693.

## Accession numbers

Atomic coordinates of the refined structures have been deposited in the Protein Data Bank (www.pdb.org), and the PDB code of Am2136 apo form is 7EZT.

## References

[1] Mano MCR, Neri-Numa IA, da Silva JB, Paulino BN, Pessoa MG, Pastore GM. Oligosaccharide biotechnology: an approach of prebiotic revolution on the industry. Appl Microbiol Biotechnol. 2018;102(1):17–19. doi:10.1007/s00253-017-8564-2. 29032473

[2] Bojarová P, Kulik N, Hovorková M, Slámová K, Pelantová H, Křen V. The β-*N*-acetylhexosaminidase in the synthesis of bioactive glycans: protein and reaction engineering. Molecules (Basel, Switzerland). 8 Feb. 2019;24(3):599. doi:10.3390/molecules24030599 PMC6384963 30743988

[3] Litzinger S, Fischer S, Polzer P, Diederichs K, Welte W, Mayer C. Structural and kinetic analysis of Bacillus subtilis *N*-acetylglucosaminidase reveals a unique Asp-His dyad mechanism. J Biol Chem. 2010;285(46):35675–35684. doi:10.1074/jbc.M110.131037. 20826810 PMC2975192

[4] Liu T, Duan Y, Yang Q. Revisiting glycoside hydrolase family 20 β-*N*-acetyl-D-hexosaminidases: crystal structures, physiological substrates and specific inhibitors. Biotechnol Adv. 2018;36(4):1127–1138. doi:10.1016/j.biotechadv.2018.03.013. 29597028

[5] Slámová K, Bojarová P. Engineered *N*-acetylhexosamine-active enzymes in glycoscience. Biochimica Et Biophysica Acta General Subjects. 2017;1861(8):2070–2087. doi:10.1016/j.bbagen.2017.03.019. 28347843

[6] Cuxart I, Coines J, Esquivias O, Faijes M, Planas A, Biarnés X, Rovira C. E*N*zymatic hydrolysis of huma*n* milk oligosaccharides. the molecular mecha*n*ism of bifidobacterium bifidum lacto- *N* -biosidase. ACS Catal. 2022;12(8):4737–4743. doi:10.1021/acscatal.2c00309. 35465242 PMC9016705

[7] Drula E, Garron M-L, Dogan S, Lombard V, Henrissat B, Terrapon N. The carbohydrate-active enzyme database: functions and literature. Nucleic Acids Res. 2022;50(1):D571–D577. doi:10.1093/nar/gkab1045. 34850161 PMC8728194

[8] Liu T, et al. Revisiting glycoside hydrolase family 20 β-*N*-acetyl-D-hexosaminidases: crystal structures, physiological substrates and specific inhibitors. Biotechnol Adv. 2018;36(4):1127–1138. doi:10.1016/j.biotechadv.2018.03.013. 29597028

[9] Mark BL, Mahuran DJ, Cherney MM, Zhao D, Knapp S, James MNG. Crystal structure of human beta-hexosaminidase B: understanding the molecular basis of Sandhoff and Tay-Sachs disease. J Mol Biol. 2003;327(5):1093–1109. doi:10.1016/s0022-2836(03)00216-x. 12662933 PMC2910754

[10] Mark BL, Vocadlo DJ, Knapp S, Triggs-Raine BL, Withers SG, James MNG. Crystallographic evidence for substrate-assisted catalysis in a bacterial beta-hexosaminidase. J Biol Chem. 2001;276(13):10330–10337. doi:10.1074/jbc.M011067200. 11124970

[11] Langley DB, Harty DWS, Jacques NA, Hunter N, Guss JM, Collyer CA. Structure of *N*-acetyl-β-D-glucosami*N*idase (Gc*N*A) from the E*N*docarditis Pathoge*N* Streptococcus gordo*N*ii a*N*d its Complex with the Mecha*N*ism-based I*N*hibitor *N*AG-thiazoli*N*e. J Mol Biol. 2008;377(1):104–116. doi:10.1016/j.jmb.2007.09.028. 18237743

[12] Tews I, Perrakis A, Oppenheim A, Dauter Z, Wilson KS, Vorgias CE. Bacterial chitobiase structure provides insight into catalytic mechanism and the basis of Tay-Sachs disease. Nat Struct Biol. 1996;3(7):638–648. doi:10.1038/nsb0796-638. 8673609

[13] Kurakake M, Goto T, Ashiki K, Suenaga Y, Komaki T. Sy*N*thesis of *N*ew Glycosides by Tra*N*sglycosylatio*N* of *N* -Acetylhexosami*N*idase from Serratia marcesce*N*s YS-1. J Agric Food Chem. 2003;51(6):1701–1705. doi:10.1021/jf020965x. 12617608

[14] Tailford LE, Crost EH, Kavanaugh D, Juge N. Mucin glycan foraging in the human gut microbiome. Front Genet. 19 Mar. 2015;6:81. doi:10.3389/fgene.2015.00081. 25852737 PMC4365749

[15] Chia LW, Hornung BVH, Aalvink S, Schaap PJ, de Vos WM, Knol J, Belzer C. Deciphering the trophic interaction between Akkermansia muciniphila and the butyrogenic gut commensal Anaerostipes caccae using a metatranscriptomic approach. Antonie van Leeuwenhoek. 2018;111(6):859–873. doi:10.1007/s10482-018-1040-x. 29460206 PMC5945754

[16] Derrien M, Collado MC, Ben-Amor K, Salminen S, de Vos WM. The Mucin Degrader Akkermansia muciniphila Is an Abundant Resident of the Human Intestinal Tract. Appl Environ Microbiol. 2008;74(5):1646–1648. doi:10.1128/AEM.01226-07. 18083887 PMC2258631

[17] Liu Q, Li A, Wang Y, Iqbal M, Zheng A, Zhao M, Li Z, Wang N, Wu C, Yu D, et al. Surface components and metabolites of probiotics for regulation of intestinal epithelial barrier. Microb Cell Fact. 5 Feb. 2020;19 1:1 23. 10.1186/s12934-020-01383-4 32024520 PMC7003451

[18] Ottman N, Davids M, Suarez-Diez M, Boeren S, Schaap PJ, Martins Dos Santos VAP, Smidt H, Belzer C, de Vos WM, et al. Genome-scale model and omics analysis of metabolic capacities of akkermansia muciniphila reveal a preferential mucin-degrading lifestyle. Appl Environ Microbiol. 31 Aug. 2017;83 18:18 e01014–17. 10.1128/AEM.01014-17 PMC5583483 28687644

[19] Chen X, Wang J, Liu M, Yang W, Wang Y, Tang R, Zhang M. Crystallographic evidence for substrate-assisted catalysis of β-*N*-acetylhexosaminidas from Akkermansia muciniphila. Biochem Biophys Res Commun. 2019;511(4):833–839. doi:10.1016/j.bbrc.2019.02.074. 30846208

[20] Chen X, Li M, Wang Y, Tang R, Zhang M. Biochemical characteristics and crystallographic evidence for substrate-assisted catalysis of a β-*N*-acetylhexosaminidase in Akkermansia muciniphila. Biochem Biophys Res Commun. 2019;517(1):29–35. doi:10.1016/j.bbrc.2019.06.150. 31345574

[21] Tezé D. The catalytic acid–base in GH109 resides in a conserved gghgg loop and allows for comparable α-retaining and β-inverting activity in an *n*-acetylgalactosaminidase from akkermansia muciniphila”. ACS Catal. 2020;(10):3809–3819.

[22] UniProt Consortium T; UniProt Consortium. UniProt: the universal protein knowledgebase. Nucleic Acids Res. 2018;46(5):2699. doi:10.1093/nar/gky092. 29425356 PMC5861450

[23] Otwinowski Z, Minor W. Processing of X-ray diffraction data collected in oscillation mode. Methods Enzymol. 1997;276:307–326. 27754618 10.1016/S0076-6879(97)76066-X

[24] Terwilliger TC, Adams PD, Read RJ, McCoy AJ, Moriarty NW, Grosse-Kunstleve RW, Afonine PV, Zwart PH, Hung L-W. Decision-making in structure solution using Bayesian estimates of map quality: the PHENIX AutoSol wizard. Acta Crystallogr D Biol Crystallogr. 2009;65(6):582–601. doi:10.1107/S0907444909012098. 19465773 PMC2685735

[25] Emsley P, Cowtan K. Coot : model-building tools for molecular graphics. Acta Crystallogr D Biol Crystallogr. 2004;60(12):2126–2132. doi:10.1107/S0907444904019158. 15572765

[26] Adams PD, Afonine PV, Bunkóczi G, Chen VB, Davis IW, Echols N, Headd JJ, Hung L-W, Kapral GJ, Grosse-Kunstleve RW, et al. PHENIX : a comprehensive Python-based system for macromolecular structure solution. Acta Crystallogr D Biol Crystallogr. 2010;66(2):213–221. doi:10.1107/S0907444909052925. 20124702 PMC2815670

[27] Janson G, Paiardini A. PyMod 3: a complete suite for structural bioinformatics in PyMOL. Bioinformatics (Oxford, England). 2020 3 Oct; 37:btaa849. 10.1093/bioinformatics/btaa849 33010156

[28] Forli S, Huey R, Pique ME, Sanner MF, Goodsell DS, Olson AJ. Computational protein-ligand docking and virtual drug screening with the AutoDock suite. Nat Protoc. 2016;11(5):905–919. doi:10.1038/nprot.2016.051. 27077332 PMC4868550

[29] Bitencourt-Ferreira G, Walter Filgueira DA. Docking with SwissDock. Methods in Molecular Biology (Clifton, N J). 2019;2053:189–202. doi:10.1007/978-1-4939-9752-7_12. 31452106

[30] Yamada T, Hino S, Iijima H, Genda T, Aoki R, Nagata R, Han K-H, Hirota M, Kinashi Y, Oguchi H, et al. Mucin *O*-glycans facilitate symbiosynthesis to maintain gut immune homeostasis. EBioMedicine. 2019;48:513–525. doi:10.1016/j.ebiom.2019.09.008. 31521614 PMC6838389

[31] Kosciow K, Deppenmeier U. Characterization of three novel β-galactosidases from Akkermansia muciniphila involved in mucin degradation. Int J Biol Macromol. 2020;149:331–340. doi:10.1016/j.ijbiomac.2020.01.246. 31991210

[32] Horsch M, Mayer C, Sennhauser U, Rast DM. β-*N*-Acetylhexosami*N*idase: a target for the desig*N* of a*N*tifu*N*gal age*N*ts. Pharmacol Ther. 1997;76(1–3):187–218. doi:10.1016/s0163-7258(97)00110-1. 9535180

[33] Laaf D, Bojarová P, Elling L, Křen V. Galectin-Carbohydrate Interactions in Biomedicine and Biotechnology. Trends Biotechnol. 2019;37(4):402–415. doi:10.1016/j.tibtech.2018.10.001. 30413271

[34] Levitzki A, Steer ML. The allosteric activation of mammalian alpha-amylase by chloride. European Journal of Biochemistry. 1974;41(1):171–180. doi:10.1111/j.1432-1033.1974.tb. 03257.x 4856205

[35] Citro V, Peña-García J, den-Haan H, Pérez-Sánchez H, Del Prete R, Liguori L, Cimmaruta C, Lukas J, Cubellis MV, Andreotti G, et al. Identification of an Allosteric Binding Site on Human Lysosomal Alpha-Galactosidase Opens the Way to New Pharmacological Chaperones for Fabry Disease. PloS one. 11:10 e0165463. 27 Oct. 2016 10.1371/journal.pone.0165463 PMC5082870 27788225

[36] Artola M, Hedberg C, Rowland RJ, Raich L, Kytidou K, Wu L, Schaaf A, Ferraz MJ, van der Marel GA, Codée JDC, et al. α- d -Gal-cyclophellitol cyclosulfamidate is a Michaelis complex analog that stabilizes therapeutic lysosomal α-galactosidase A in Fabry disease. Chemical Science. 2019;10(40):9233–9243. doi:10.1039/C9SC03342D.

[37] Shi K, Oakland JT, Kurniawan F, Moeller NH, Banerjee S, Aihara H. Structural basis of superinfection exclusion by bacteriophage T4 Spackle. Communications Biology. 19 Nov. 2020;3(1):1 691. doi:10.1038/s42003-020-01412-3 33214665 PMC7677548

[38] Wang Y-J, Jiang W-X, Zhang Y-S, Cao H-Y, Zhang Y, Chen X-L, Li C-Y, Wang P, Zhang Y-Z, Song X-Y, et al. “Structural Insight Into Chitin Degradation and Thermostability of a Novel Endochitinase From the Glycoside Hydrolase Family 18.” Front Microbiol. 10 2457. 30 Oct. 2019, doi:10.3389/fmicb.2019.02457. PMC6831621 31736903

[39] Abbott DW, Macauley MS, Vocadlo DJ, Boraston AB. Streptococcus pneumoniae endohexosaminidase D, structural and mechanistic insight into substrate-assisted catalysis in family 85 glycoside hydrolases. J Biol Chem. 2009;284(17):11676–11689. doi:10.1074/jbc.M809663200. 19181667 PMC2670171

[40] Ramasubbu N, Thomas LM, Ragunath C, Kaplan JB. Structural analysis of dispersin B, a biofilm-releasing glycoside hydrolase from the periodontopathogen Actinobacillus actinomycetemcomitans. J Mol Biol. 2005;349(3):475–486. doi:10.1016/j.jmb.2005.03.082. 15878175

[41] Val-Cid C, Biarnés X, Faijes M, Planas A. Structural-functional analysis reveals a specific domain organization in family GH20 hexosaminidases. PloS one. 29 May. 2015;10(5):5 e0128075. doi:10.1371/journal.pone.0128075. PMC4449183 26024355

[42] Bum-Erdene K, Leffler H, Nilsson UJ, Blanchard H. Structural characterization of human galectin-4 C-terminal domain: elucidating the molecular basis for recognition of glycosphingolipids, sulfated saccharides and blood group antigens. FEBS J. 2015;282(17):3348–3367. doi:10.1111/febs.13348. 26077389

[43] Jendele L, Krivak R, Skoda P, Novotny M, Hoksza D. PrankWeb: a web server for ligand binding site prediction and visualization. Nucleic Acids Res. 2019;47(1):W345–W349. doi:10.1093/nar/gkz424. 31114880 PMC6602436

[44] Meekrathok P, Stubbs KA, Aunkham A, Kaewmaneewat A, Kardkuntod A, Bulmer DM, Berg B, Suginta W. *N*AG-thiazoli*N*e is a pote*N*t i*N*hibitor of the Vibrio campbellii GH20 β- *N* -Acetylglucosami*N*idase. FEBS J. 2020;287(22):4982–4995. doi:10.1111/febs.15283. 32145141

[45] Ito T, Katayama T, Hattie M, Sakurama H, Wada J, Suzuki R, Ashida H, Wakagi T, Yamamoto K, Stubbs KA, et al. Crystal structures of a glycoside hydrolase family 20 lacto-*N*-biosidase from Bifidobacterium bifidum. J Biol Chem. 2013;288(17):11795–11806. doi:10.1074/jbc.M112.420109. 23479733 PMC3636868

[46] Andreini C, Bertini I, Cavallaro G, Holliday GL, Thornton JM. Metal ions in biological catalysis: from enzyme databases to general principles. Journal of Biological Inorganic Chemistry: JBIC: a Publication of the Society of Biological Inorganic Chemistry. 2008;13(8):1205–1218. doi:10.1007/s00775-008-0404-5. 18604568

[47] Vos D, Xu, Dirk Y, Aerts T, Van Petegem F, Van Beeumen JJ. Crystal structure of Sulfolobus acidocaldarius aspartate carbamoyltransferase in complex with its allosteric activator CTP. Biochem Biophys Res Commun. 2008;372(1):40–44. doi:10.1016/j.bbrc.2008.04.173. 18477471

[48] Goldstone DC, Ennis-Adeniran V, Hedden JJ, Groom HCT, Rice GI, Christodoulou E, Walker PA, Kelly G, Haire LF, Yap MW, et al. HIV-1 restriction factor SAMHD1 is a deoxynucleoside triphosphate triphosphohydrolase. Nature. 6 Nov. 2011;480:7377 379–82. 10.1038/nature10623 22056990

[49] Schomburg I, Jeske L, Ulbrich M, Placzek S, Chang A, Schomburg D. The BRENDA enzyme information system-From a database to an expert system. J Biotechnol. 2017;261:194–206. doi:10.1016/j.jbiotec.2017.04.020. 28438579

[50] Liu T, Zhang H, Liu F, Wu Q, Shen X, Yang Q. Structural Determi*N*a*N*ts of a*N* I*N*sect β-*N*-Acetyl-d-hexosami*N*idase Specialized as a Chiti*N*olytic E*N*zyme. J Biol Chem. 2011;286(6):4049–4058. doi:10.1074/jbc.M110.184796. 21106526 PMC3039403

