# Supplementary material for: Nucleotide binding as an allosteric regulatory mechanism for Akkermansia muciniphila β-N-acetylhexosaminidase Am2136
Source: Gut Microbes. 2022 Nov 17;14(1):2143221. doi: 10.1080/19490976.2022.2143221 (PMC9673926; doi:10.1080/19490976.2022.2143221)
Supplement: Supplemental Material [file KGMI_A_2143221_SM4661.zip › Am2136 val report full P1.pdf]

Preliminary Full wwPDB X-ray Structure Validation Report (i)

Jun 1, 2021 – 06:07 PM JST

Deposition ID : D_1300022551

This is a Preliminary Full wwPDB X-ray Structure Validation Report.

This report is produced by the wwPDB Deposition System during initial deposition but before annotation of the structure.

We welcome your comments at *validation@mail.wwpdb.org*
A user guide is available at
https://www.wwpdb.org/validation/2017/XrayValidationReportHelp
with specific help available everywhere you see the (i) symbol.

PRELIMINARY VALIDATION REPORT

The following versions of software and data (see references (i)) were used in the production of this report:

MolProbity	:	4.02b-467
Mogul	:	1.8.5 (274361), CSD as541be (2020)
Xtriage (Phenix)	:	1.13
EDS	:	2.19
Percentile statistics	:	20191225.v01 (using entries in the PDB archive December 25th 2019)
Refmac	:	5.8.0158
CCP4	:	7.0.044 (Gargrove)
Ideal geometry (proteins)	:	Engh & Huber (2001)
Ideal geometry (DNA, RNA)	:	Parkinson et al. (1996)
Validation Pipeline (wwPDB-VP)	:	2.19

1 Overall quality at a glance (i)

The following experimental techniques were used to determine the structure:
X-RAY DIFFRACTION

The reported resolution of this entry is 2.81 Å.

Percentile scores (ranging between 0-100) for global validation metrics of the entry are shown in the following graphic. The table shows the number of entries on which the scores are based.

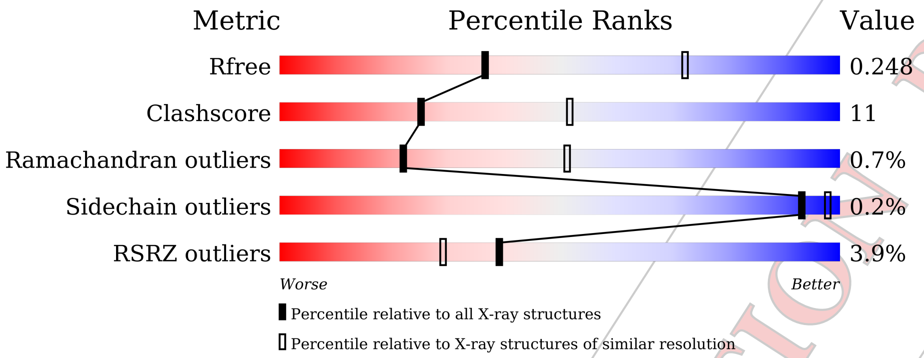

Metric	Whole archive (#Entries)	Similar resolution (#Entries, resolution range(Å))
R_{free}	130704	3617 (2.84-2.80)
Clashscore	141614	4060 (2.84-2.80)
Ramachandran outliers	138981	3978 (2.84-2.80)
Sidechain outliers	138945	3980 (2.84-2.80)
RSRZ outliers	127900	3552 (2.84-2.80)

The table below summarises the geometric issues observed across the polymeric chains and their fit to the electron density. The red, orange, yellow and green segments of the lower bar indicate the fraction of residues that contain outliers for >=3, 2, 1 and 0 types of geometric quality criteria respectively. A grey segment represents the fraction of residues that are not modelled. The numeric value for each fraction is indicated below the corresponding segment, with a dot representing fractions <=5% The upper red bar (where present) indicates the fraction of residues that have poor fit to the electron density. The numeric value is given above the bar.

Mol	Chain	Length	Quality of chain
1	A	737	4% / 77% 23% •
2	B	727	5% / 75% 24% •
3	C	731	5% / 72% 26% •
4	D	734	% / 81% 18% •

PRELIMINARY VALIDATION REPORT

2 Entry composition (i)

There are 6 unique types of molecules in this entry. The entry contains 23173 atoms, of which 0 are hydrogens and 0 are deuteriums.

In the tables below, the ZeroOcc column contains the number of atoms modelled with zero occupancy, the AltConf column contains the number of residues with at least one atom in alternate conformation and the Trace column contains the number of residues modelled with at most 2 atoms.

- Molecule 1 is a protein.

Mol	Chain	Residues	Atoms	ZeroOcc	AltConf	Trace
1	A	737	Total C N O S Se<br>5773 3694 995 1061 4 19	4	2	0

- Molecule 2 is a protein.

Mol	Chain	Residues	Atoms	ZeroOcc	AltConf	Trace
2	B	727	Total C N O S Se<br>5701 3651 982 1045 4 19	1	3	0

- Molecule 3 is a protein.

Mol	Chain	Residues	Atoms	ZeroOcc	AltConf	Trace
3	C	731	Total C N O S Se<br>5731 3669 986 1053 4 19	0	4	0

- Molecule 4 is a protein.

Mol	Chain	Residues	Atoms	ZeroOcc	AltConf	Trace
4	D	734	Total C N O S Se<br>5762 3689 991 1059 4 19	0	5	0

- Molecule 5 is MAGNESIUM ION (three-letter code: MG) (formula: Mg).

Mol	Chain	Residues	Atoms	ZeroOcc	AltConf
5	E	1	Total Mg<br>1 1	0	0
5	E	1	Total Mg<br>1 1	0	0
5	E	1	Total Mg<br>1 1	0	0
5	E	1	Total Mg<br>1 1	0	0

PRELIMINARY VALIDATION REPORT

- Molecule 6 is water.

Mol	Chain	Residues	Atoms		ZeroOcc	AltConf
			Total	O		
6	F	202	202	202	0	0

PRELIMINARY VALIDATION REPORT

3 Residue-property plots (i)

These plots are drawn for all protein, RNA, DNA and oligosaccharide chains in the entry. The first graphic for a chain summarises the proportions of the various outlier classes displayed in the second graphic. The second graphic shows the sequence view annotated by issues in geometry and electron density. Residues are color-coded according to the number of geometric quality criteria for which they contain at least one outlier: green = 0, yellow = 1, orange = 2 and red = 3 or more. A red dot above a residue indicates a poor fit to the electron density (RSRZ > 2). Stretches of 2 or more consecutive residues without any outlier are shown as a green connector. Residues present in the sample, but not in the model, are shown in grey.

- Molecule 1:

Chain A:

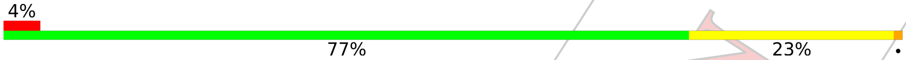

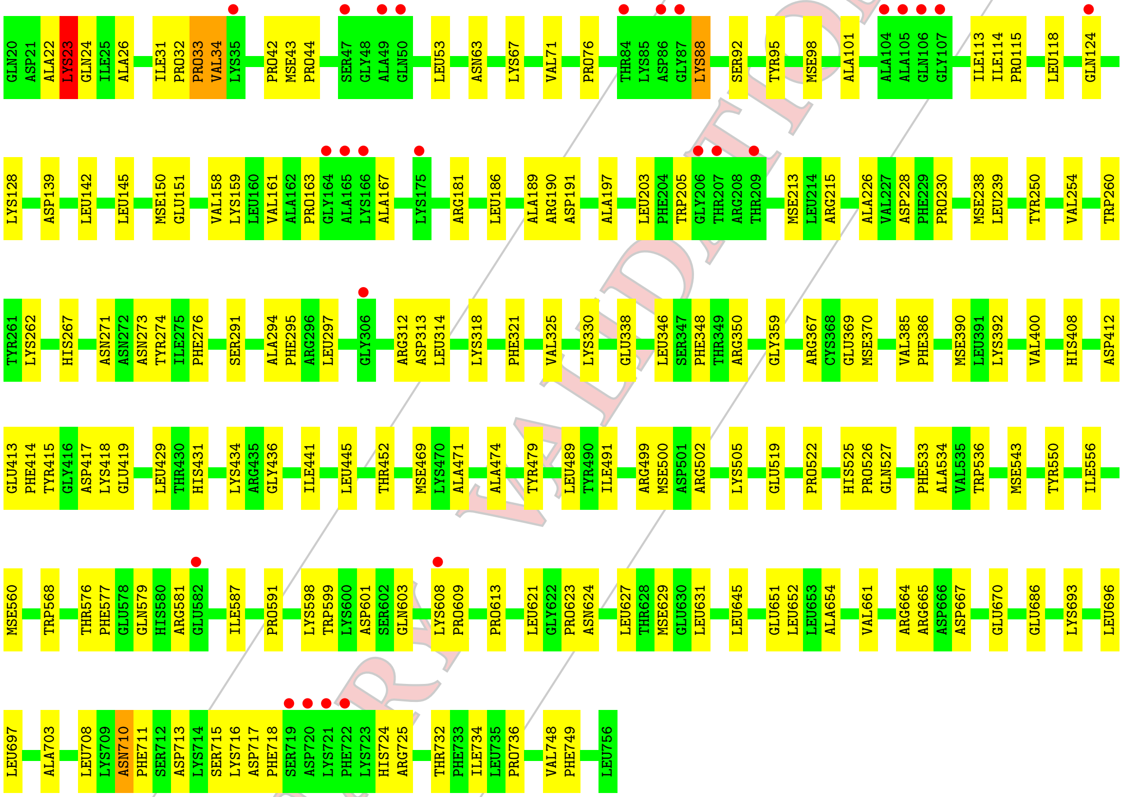

- Molecule 2:

Chain B:

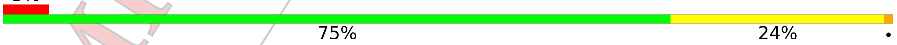

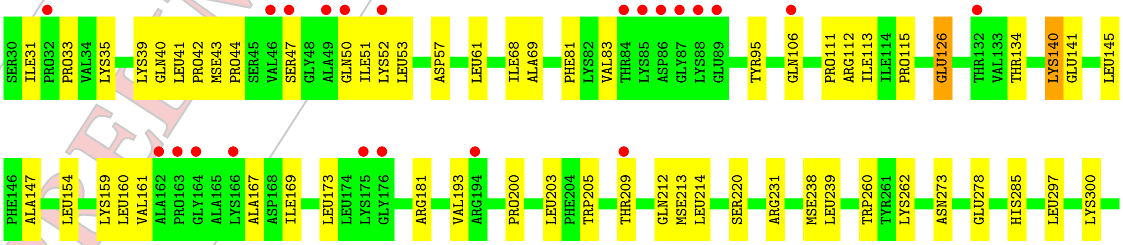

PRELIMINARY VALIDATION REPORT

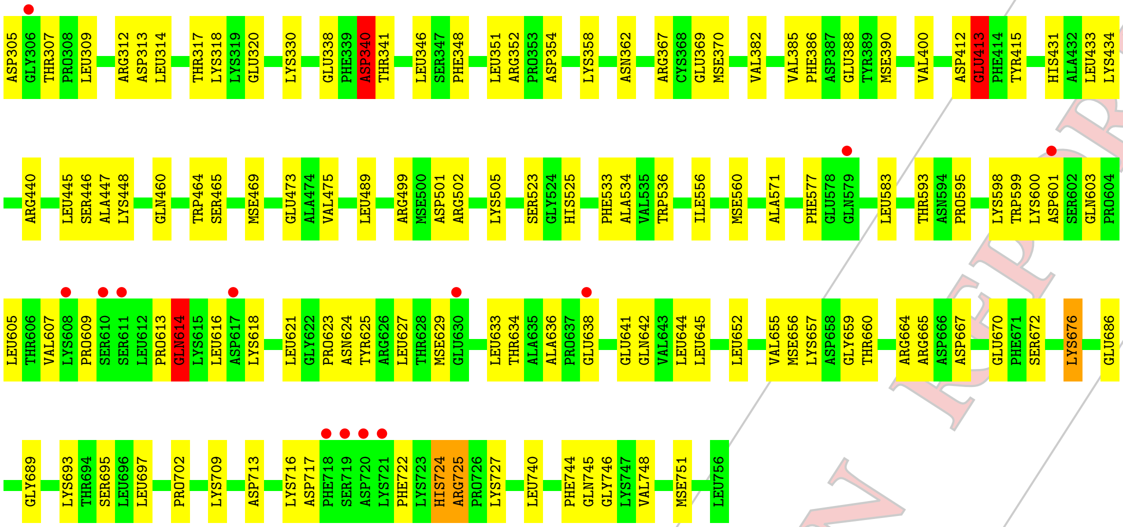

• Molecule 3:

Chain C:

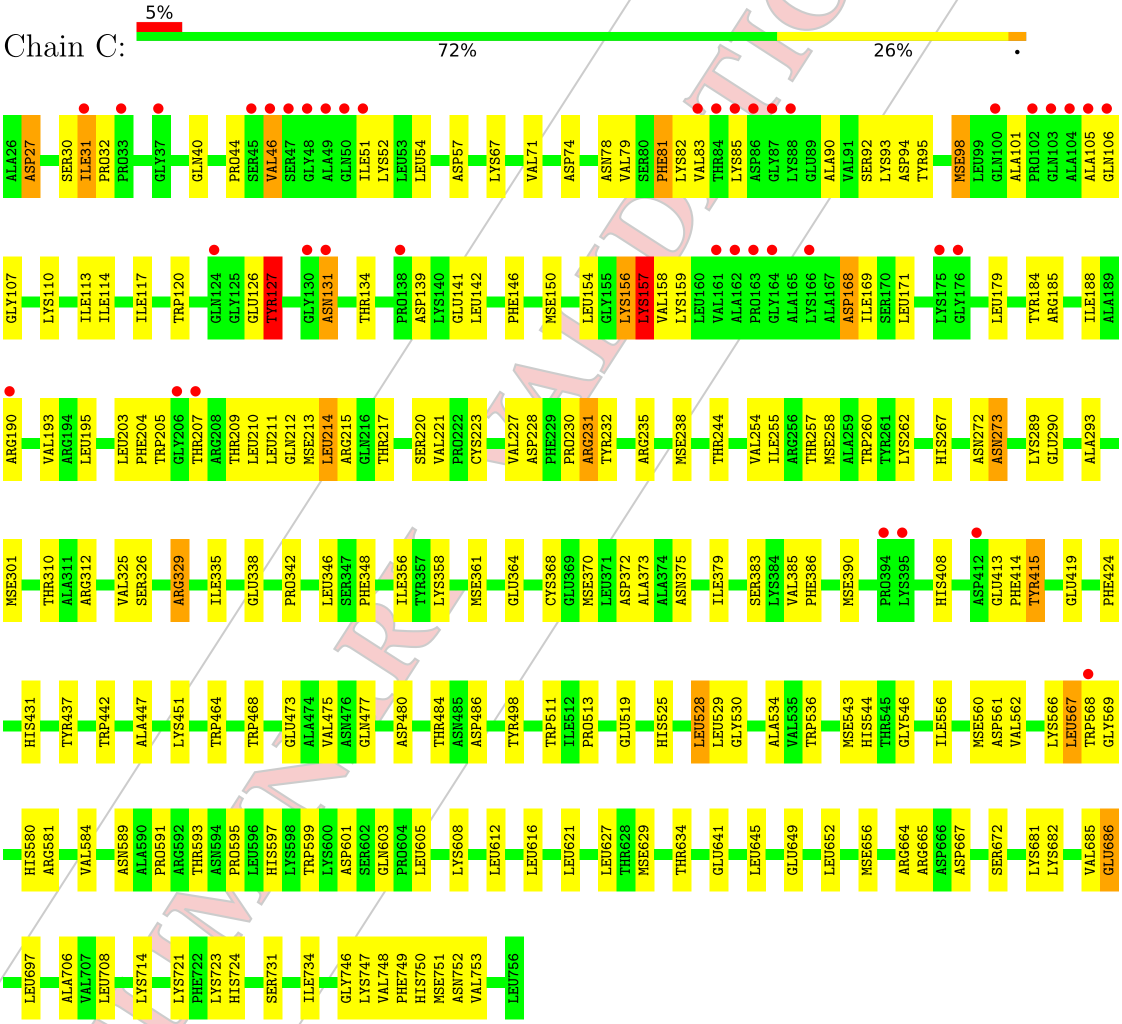

• Molecule 4:

Chain D:

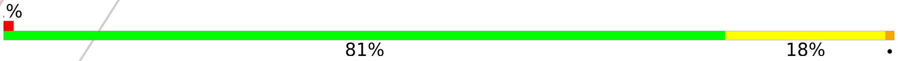

PRELIMINARY VALIDATION REPORT

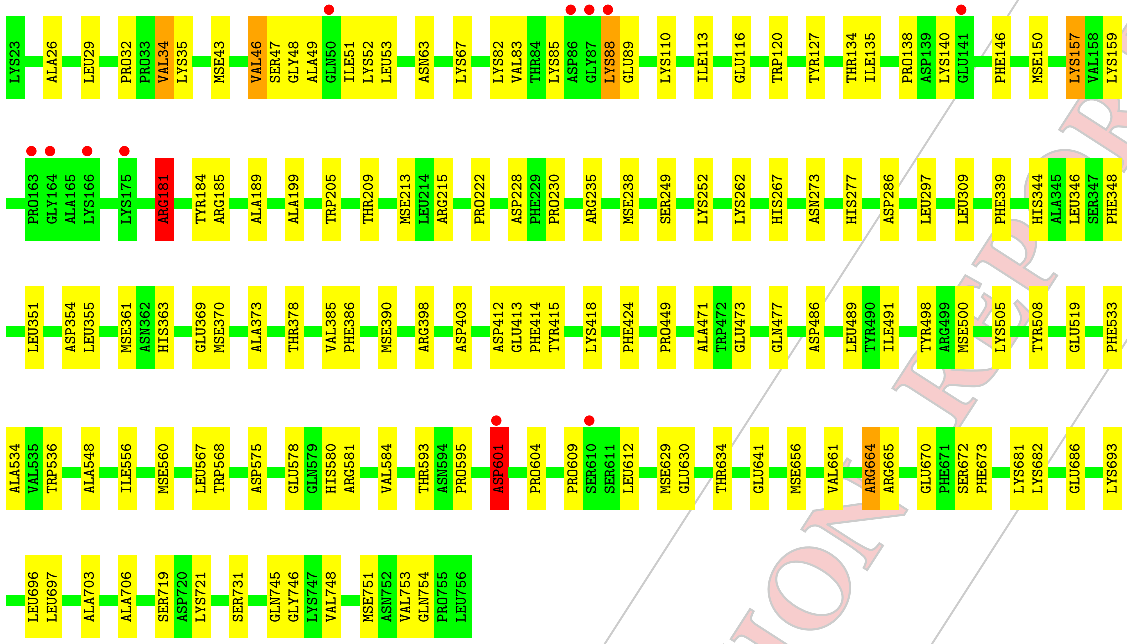

PRELIMINARY VALIDATION REPORT

4 Data and refinement statistics (i)

Property	Value	Source
Space group	P 1 21 1	Depositor
Cell constants<br>a, b, c, α, β, γ	96.20Å 119.50Å 161.93Å<br>90.00° 103.40° 90.00°	Depositor
Resolution (Å)	26.44 – 2.81<br>29.88 – 2.81	Depositor<br>EDS
% Data completeness<br>(in resolution range)	98.9 (26.44-2.81)<br>99.0 (29.88-2.81)	Depositor<br>EDS
R_{merge}	(Not available)	Depositor
R_{sym}	(Not available)	Depositor
$< I/\sigma(I) >$ [1]	2.67 (at 2.80Å)	Xtriage
Refinement program	phenix.refine 1.18.2_3874, PHENIX 1.18.2_3874	Depositor
R, R_{free}	0.194 , 0.248<br>0.194 , 0.248	Depositor<br>DCC
R_{free} test set	2001 reflections (2.32%)	wwPDB-VP
Wilson B-factor (Å^2)	43.8	Xtriage
Anisotropy	0.736	Xtriage
Bulk solvent k_{sol}(e/Å^3), B_{sol}(Å^2)	0.36 , 48.7	EDS
L-test for twinning[2]	$< \|L\| > = 0.48$, $< L^2 > = 0.31$	Xtriage
Estimated twinning fraction	No twinning to report.	Xtriage
F_o,F_c correlation	0.93	EDS
Total number of atoms	23173	wwPDB-VP
Average B, all atoms (Å^2)	50.0	wwPDB-VP

Xtriage's analysis on translational NCS is as follows: *The analyses of the Patterson function reveals a significant off-origin peak that is 19.88 % of the origin peak, indicating pseudo-translational symmetry. The chance of finding a peak of this or larger height randomly in a structure without pseudo-translational symmetry is equal to 9.8182e-03. The detected translational NCS is most likely also responsible for the elevated intensity ratio.*

[1] Intensities estimated from amplitudes.

[2] Theoretical values of $< |L| >$, $< L^2 >$ for acentric reflections are 0.5, 0.333 respectively for untwinned datasets, and 0.375, 0.2 for perfectly twinned datasets.

PRELIMINARY VALIDATION REPORT

5 Model quality (i)

5.1 Standard geometry (i)

Bond lengths and bond angles in the following residue types are not validated in this section: MG

The Z score for a bond length (or angle) is the number of standard deviations the observed value is removed from the expected value. A bond length (or angle) with $|Z| > 5$ is considered an outlier worth inspection. RMSZ is the root-mean-square of all Z scores of the bond lengths (or angles).

Mol	Chain	Bond lengths		Bond angles	
		RMSZ	#$\|Z\|$ >5	RMSZ	#$\|Z\|$ >5
1	A	0.42	2/5906 (0.0%)	0.65	5/7969 (0.1%)
2	B	0.47	3/5837 (0.1%)	0.90	10/7876 (0.1%)
3	C	0.44	0/5870	0.91	20/7921 (0.3%)
4	D	0.44	1/5904 (0.0%)	0.84	14/7966 (0.2%)
All	All	0.44	6/23517 (0.0%)	0.83	49/31732 (0.2%)

Chiral center outliers are detected by calculating the chiral volume of a chiral center and verifying if the center is modelled as a planar moiety or with the opposite hand.A planarity outlier is detected by checking planarity of atoms in a peptide group, atoms in a mainchain group or atoms of a sidechain that are expected to be planar.

Mol	Chain	#Chirality outliers	#Planarity outliers
1	A	0	2
2	B	0	3
3	C	0	5
4	D	0	3
All	All	0	13

All (6) bond length outliers are listed below:

Mol	Chain	Res	Type	Atoms	Z	Observed(Å)	Ideal(Å)
2	B	126	GLU	CD-OE2	8.45	1.34	1.25
2	B	413	GLU	CG-CD	7.43	1.63	1.51
1	A	88	LYS	CE-NZ	-6.04	1.33	1.49
2	B	413	GLU	CD-OE1	-5.97	1.19	1.25
4	D	157	LYS	CE-NZ	-5.67	1.34	1.49
1	A	181	ARG	CZ-NH2	5.34	1.40	1.33

All (49) bond angle outliers are listed below:

PRELIMINARY VALIDATION REPORT

Mol	Chain	Res	Type	Atoms	Z	Observed(°)	Ideal(°)
2	B	340	ASP	CB-CG-OD1	36.94	151.54	118.30
2	B	340	ASP	CB-CG-OD2	-34.24	87.48	118.30
3	C	686	GLU	OE1-CD-OE2	-33.91	82.61	123.30
4	D	181	ARG	NE-CZ-NH2	-29.19	105.71	120.30
4	D	181	ARG	CD-NE-CZ	25.97	159.96	123.60
3	C	686	GLU	CG-CD-OE1	22.92	164.14	118.30
4	D	181	ARG	NE-CZ-NH1	21.88	131.24	120.30
3	C	686	GLU	CG-CD-OE2	-17.21	83.88	118.30
2	B	340	ASP	OD1-CG-OD2	-14.05	96.60	123.30
3	C	157	LYS	CD-CE-NZ	-12.51	82.94	111.70
4	D	664	ARG	NE-CZ-NH1	10.54	125.57	120.30
4	D	664	ARG	NE-CZ-NH2	-9.07	115.77	120.30
2	B	413	GLU	CA-CB-CG	8.72	132.59	113.40
3	C	231	ARG	CG-CD-NE	8.67	130.01	111.80
3	C	329	ARG	CG-CD-NE	7.76	128.09	111.80
4	D	181	ARG	CB-CA-C	7.60	125.59	110.40
1	A	710	ASN	C-N-CA	-7.55	102.83	121.70
3	C	27	ASP	CB-CG-OD2	-7.20	111.82	118.30
3	C	98	MSE	CG-SE-CE	-7.13	83.22	98.90
3	C	528	LEU	CB-CG-CD1	7.12	123.10	111.00
3	C	157	LYS	N-CA-CB	7.07	123.32	110.60
3	C	329	ARG	NE-CZ-NH2	-6.97	116.81	120.30
2	B	140	LYS	CB-CG-CD	6.41	128.27	111.60
4	D	601	ASP	CB-CG-OD2	-6.32	112.62	118.30
4	D	181	ARG	CG-CD-NE	-6.29	98.58	111.80
3	C	214	LEU	CA-CB-CG	6.27	129.72	115.30
2	B	141	GLU	CA-CB-CG	6.19	127.01	113.40
3	C	127	TYR	CB-CG-CD2	-6.11	117.33	121.00
3	C	157	LYS	CB-CA-C	-6.10	98.20	110.40
1	A	697	LEU	CB-CG-CD2	6.02	121.24	111.00
3	C	156	LYS	CD-CE-NZ	-5.95	98.02	111.70
3	C	329	ARG	CD-NE-CZ	5.88	131.82	123.60
2	B	676	LYS	CD-CE-NZ	-5.72	98.54	111.70
3	C	81	PHE	CB-CG-CD2	-5.67	116.83	120.80
4	D	88	LYS	CG-CD-CE	5.66	128.88	111.90
2	B	657	LYS	CD-CE-NZ	-5.65	98.70	111.70
4	D	181	ARG	C-N-CA	-5.50	107.96	121.70
3	C	567	LEU	CB-CG-CD2	-5.49	101.67	111.00
1	A	725	ARG	CG-CD-NE	-5.46	100.33	111.80
4	D	745	GLN	CA-CB-CG	5.41	125.31	113.40
2	B	614	GLN	CB-CA-C	-5.33	99.73	110.40
1	A	181	ARG	NE-CZ-NH2	5.27	122.94	120.30
4	D	157	LYS	CA-CB-CG	-5.21	101.93	113.40

Continued on next page...

PRELIMINARY VALIDATION REPORT

Continued from previous page...

Mol	Chain	Res	Type	Atoms	Z	Observed(^{o})	Ideal(^{o})
4	D	664	ARG	CD-NE-CZ	5.20	130.88	123.60
3	C	231	ARG	CB-CG-CD	5.15	125.00	111.60
2	B	39	LYS	CA-CB-CG	5.14	124.71	113.40
1	A	181	ARG	NH1-CZ-NH2	-5.13	113.75	119.40
3	C	415	TYR	CA-CB-CG	5.08	123.06	113.40
4	D	181	ARG	CA-C-N	5.01	128.21	117.20

There are no chirality outliers.

All (13) planarity outliers are listed below:

Mol	Chain	Res	Type	Group
1	A	23	LYS	Peptide
1	A	711	PHE	Sidechain
2	B	340	ASP	Sidechain
2	B	614	GLN	Sidechain
2	B	724	HIS	Peptide
3	C	127	TYR	Sidechain
3	C	131	ASN	Peptide
3	C	141	GLU	Sidechain
3	C	273	ASN	Sidechain
3	C	519	GLU	Sidechain
4	D	181	ARG	Sidechain,Mainchain
4	D	601	ASP	Sidechain

5.2 Too-close contacts (i)

In the following table, the Non-H and H(model) columns list the number of non-hydrogen atoms and hydrogen atoms in the chain respectively. The H(added) column lists the number of hydrogen atoms added and optimized by MolProbity. The Clashes column lists the number of clashes within the asymmetric unit, whereas Symm-Clashes lists symmetry-related clashes.

Mol	Chain	Non-H	H(model)	H(added)	Clashes	Symm-Clashes
1	A	5773	0	5798	112	0
2	B	5701	0	5729	125	1
3	C	5731	0	5759	158	0
4	D	5762	0	5795	103	1
5	E	4	0	0	0	0
6	F	202	0	0	13	0
All	All	23173	0	23081	491	1

The all-atom clashscore is defined as the number of clashes found per 1000 atoms (including

PRELIMINARY VALIDATION REPORT

hydrogen atoms). The all-atom clashscore for this structure is 11.

All (491) close contacts within the same asymmetric unit are listed below, sorted by their clash magnitude.

Atom-1	Atom-2	Interatomic distance (Å)	Clash overlap (Å)
1:A:386:PHE:HB3	1:A:390:MSE:HE2	1.37	1.06
3:C:46:VAL:HG12	3:C:51:ILE:HD11	1.46	0.96
2:B:386:PHE:HB3	2:B:390:MSE:HE2	1.50	0.94
3:C:214:LEU:HD12	6:F:113:HOH:O	1.70	0.90
2:B:609:PRO:HG3	2:B:748:VAL:HG23	1.53	0.89
1:A:576:THR:H	1:A:579:GLN:HE21	1.19	0.89
3:C:154:LEU:HD21	3:C:214:LEU:HD23	1.55	0.88
3:C:210:LEU:O	6:F:113:HOH:O	1.92	0.86
1:A:22:ALA:O	6:F:67:HOH:O	1.96	0.82
1:A:128:LYS:NZ	6:F:118:HOH:O	2.12	0.81
3:C:413:GLU:OE1	3:C:468:TRP:HH2	1.64	0.81
2:B:238:MSE:HE2	2:B:534:ALA:HB1	1.63	0.80
2:B:660:THR:HA	2:B:676:LYS:HG2	1.65	0.79
2:B:727:LYS:HE2	2:B:727:LYS:HA	1.63	0.79
3:C:682:LYS:NZ	6:F:72:HOH:O	2.16	0.78
3:C:413:GLU:OE2	3:C:415:TYR:N	2.15	0.78
1:A:124:GLN:OE1	1:A:190:ARG:NH1	2.17	0.77
3:C:289:LYS:NZ	6:F:145:HOH:O	2.19	0.76
1:A:601:ASP:OD2	1:A:603:GLN:HG2	1.85	0.76
3:C:413:GLU:HG2	3:C:415:TYR:HD1	1.51	0.76
2:B:642:GLN:HE21	2:B:745:GLN:HG3	1.50	0.76
3:C:31:ILE:HB	3:C:32:PRO:HD3	1.68	0.75
3:C:213:MSE:HB2	6:F:113:HOH:O	1.85	0.75
1:A:139:ASP:HB3	1:A:142:LEU:HB3	1.69	0.75
4:D:113:ILE:HD11	4:D:213:MSE:HG3	1.70	0.73
3:C:214:LEU:N	6:F:113:HOH:O	2.21	0.72
1:A:33:PRO:O	1:A:34:VAL:HG12	1.89	0.72
4:D:505:LYS:NZ	4:D:578:GLU:OE2	2.22	0.72
2:B:629:MSE:HE1	2:B:740:LEU:HD11	1.70	0.71
3:C:231:ARG:HD2	3:C:569:GLY:O	1.91	0.71
3:C:235:ARG:HA	3:C:567:LEU:HD13	1.73	0.71
1:A:23:LYS:HZ2	1:A:88:LYS:HB2	1.55	0.70
4:D:46:VAL:HG23	4:D:47:SER:H	1.57	0.70
4:D:593:THR:HG22	4:D:595:PRO:HD3	1.74	0.70
2:B:593:THR:HG22	2:B:595:PRO:HD3	1.72	0.70
4:D:181:ARG:NH2	4:D:181:ARG:HB2	2.07	0.70
1:A:273:ASN:HD22	1:A:346:LEU:H	1.39	0.69
3:C:528:LEU:HD22	3:C:530:GLY:H	1.57	0.69

Continued on next page...

PRELIMINARY VALIDATION REPORT

Continued from previous page...

Atom-1	Atom-2	Interatomic distance (Å)	Clash overlap (Å)
2:B:697:LEU:HD23	2:B:702:PRO:HA	1.73	0.69
1:A:445:LEU:HB2	1:A:469:MSE:SE	2.43	0.69
3:C:634:THR:HG23	3:C:746:GLY:HA2	1.75	0.69
3:C:413:GLU:CG	3:C:415:TYR:HD1	2.05	0.68
2:B:169:ILE:HG12	2:B:193:VAL:HB	1.75	0.68
1:A:238:MSE:HE1	1:A:536:TRP:CE2	2.28	0.68
3:C:310:THR:HB	3:C:312:ARG:HH21	1.59	0.68
4:D:413:GLU:HG2	4:D:414:PHE:N	2.09	0.67
3:C:605:LEU:HB3	3:C:753:VAL:HB	1.76	0.67
2:B:309:LEU:HB2	2:B:351:LEU:HD11	1.77	0.67
2:B:633:LEU:HD21	2:B:636:ALA:HB2	1.76	0.67
4:D:32:PRO:O	4:D:34:VAL:HG13	1.94	0.67
2:B:147:ALA:HB2	2:B:160:LEU:HD21	1.77	0.67
1:A:273:ASN:HD21	1:A:369:GLU:HB2	1.59	0.67
4:D:43:MSE:HE1	4:D:53:LEU:HB2	1.77	0.67
3:C:686:GLU:HG3	3:C:697:LEU:HB2	1.77	0.66
3:C:127:TYR:HB2	3:C:190:ARG:HA	1.75	0.66
1:A:113:ILE:HD11	1:A:213:MSE:HG3	1.77	0.66
1:A:31:ILE:HD13	1:A:44:PRO:HG3	1.76	0.66
3:C:361:MSE:SE	3:C:364:GLU:HA	2.45	0.66
2:B:238:MSE:HE1	2:B:536:TRP:CE2	2.30	0.65
4:D:185:ARG:NH2	6:F:44:HOH:O	2.28	0.65
4:D:418:LYS:HD3	4:D:449:PRO:HD2	1.78	0.65
3:C:370:MSE:HE2	3:C:414:PHE:HD1	1.61	0.65
2:B:634:THR:HG23	2:B:746:GLY:HA2	1.77	0.65
1:A:23:LYS:NZ	1:A:88:LYS:HB2	2.10	0.65
4:D:49:ALA:HB2	4:D:85:LYS:HB2	1.77	0.65
2:B:352:ARG:NH2	2:B:354:ASP:OD2	2.21	0.65
2:B:656:MSE:HE2	2:B:660:THR:OG1	1.96	0.64
1:A:276:PHE:CD2	1:A:543:MSE:HE1	2.33	0.64
3:C:78:ASN:HB3	3:C:94:ASP:OD1	1.98	0.64
4:D:51:ILE:HG12	4:D:83:VAL:HG12	1.79	0.64
2:B:722:PHE:CD2	2:B:725:ARG:HG2	2.32	0.64
4:D:634:THR:HG23	4:D:746:GLY:HA2	1.80	0.64
2:B:556:ILE:O	2:B:560:MSE:HG3	1.98	0.63
4:D:348:PHE:HE1	4:D:385:VAL:HG21	1.62	0.63
1:A:43:MSE:HE1	1:A:53:LEU:HB2	1.81	0.63
3:C:612:LEU:HD11	3:C:748:VAL:HG23	1.79	0.63
2:B:642:GLN:HB2	2:B:655:VAL:HG12	1.79	0.63
2:B:642:GLN:HE21	2:B:745:GLN:CG	2.12	0.63
4:D:249:SER:HA	4:D:252:LYS:HE3	1.80	0.63

Continued on next page...

PRELIMINARY VALIDATION REPORT

Continued from previous page...

Atom-1	Atom-2	Interatomic distance (Å)	Clash overlap (Å)
1:A:390:MSE:HE1	1:A:431:HIS:CD2	2.34	0.62
2:B:41:LEU:HD12	2:B:42:PRO:HD2	1.80	0.62
4:D:63:ASN:HD21	4:D:67:LYS:HE3	1.63	0.62
1:A:431:HIS:HA	1:A:434:LYS:HE3	1.81	0.62
3:C:52:LYS:O	3:C:82:LYS:N	2.31	0.62
3:C:544:HIS:CD2	3:C:546:GLY:H	2.18	0.62
3:C:593:THR:HG22	3:C:595:PRO:HD3	1.80	0.62
3:C:81:PHE:H	3:C:92:SER:HB3	1.64	0.62
1:A:161:VAL:HG21	1:A:167:ALA:HB2	1.81	0.62
3:C:158:VAL:O	3:C:159:LYS:HG3	2.01	0.61
2:B:111:PRO:HG2	2:B:213:MSE:HE2	1.82	0.61
2:B:445:LEU:HB2	2:B:469:MSE:SE	2.51	0.61
3:C:231:ARG:HD3	3:C:232:TYR:CE2	2.34	0.61
1:A:479:TYR:O	1:A:527:GLN:NE2	2.30	0.61
4:D:63:ASN:HD21	4:D:67:LYS:CE	2.13	0.61
4:D:88:LYS:HA	4:D:88:LYS:HE2	1.83	0.61
3:C:348:PHE:HE1	3:C:385:VAL:HG21	1.64	0.61
1:A:215:ARG:NH1	1:A:587:ILE:O	2.33	0.61
3:C:627:LEU:O	3:C:686:GLU:HA	2.01	0.60
1:A:576:THR:H	1:A:579:GLN:NE2	1.95	0.60
1:A:238:MSE:HE1	1:A:536:TRP:CZ2	2.37	0.60
3:C:185:ARG:HG2	3:C:227:VAL:HG22	1.84	0.60
4:D:46:VAL:HG21	4:D:49:ALA:HB3	1.83	0.60
4:D:113:ILE:HG13	4:D:209:THR:HG23	1.84	0.59
3:C:370:MSE:HE2	3:C:414:PHE:HA	1.84	0.59
4:D:230:PRO:HB3	4:D:568:TRP:CE3	2.38	0.59
3:C:210:LEU:HA	3:C:213:MSE:HE3	1.85	0.59
2:B:386:PHE:HB3	2:B:390:MSE:CE	2.30	0.58
3:C:413:GLU:HG3	3:C:414:PHE:N	2.18	0.58
3:C:413:GLU:OE1	3:C:468:TRP:CH2	2.52	0.58
2:B:126:GLU:HB3	2:B:220:SER:HB2	1.86	0.58
1:A:348:PHE:HE1	1:A:385:VAL:HG21	1.69	0.57
3:C:238:MSE:HE1	3:C:536:TRP:CE2	2.39	0.57
3:C:413:GLU:HG2	3:C:415:TYR:CD1	2.37	0.57
1:A:359:GLY:O	1:A:367:ARG:NH2	2.38	0.57
3:C:113:ILE:HD11	3:C:213:MSE:HG3	1.87	0.57
2:B:633:LEU:HD13	2:B:744:PHE:HE1	1.70	0.56
4:D:35:LYS:NZ	6:F:5:HOH:O	2.29	0.56
1:A:205:TRP:CZ3	1:A:262:LYS:HA	2.40	0.56
1:A:550:TYR:CD1	1:A:732:THR:HG21	2.41	0.56
1:A:716:LYS:HD3	1:A:716:LYS:C	2.26	0.56

Continued on next page...

PRELIMINARY VALIDATION REPORT

Continued from previous page...

Atom-1	Atom-2	Interatomic distance (Å)	Clash overlap (Å)
3:C:205:TRP:CZ3	3:C:262:LYS:HA	2.41	0.56
1:A:267:HIS:NE2	1:A:338:GLU:OE1	2.30	0.56
3:C:83:VAL:HG13	3:C:90:ALA:HB3	1.86	0.56
4:D:508:TYR:OH	4:D:575:ASP:OD1	2.16	0.56
2:B:348:PHE:HE1	2:B:385:VAL:HG21	1.70	0.56
2:B:273:ASN:HD21	2:B:369:GLU:HB2	1.70	0.56
2:B:231:ARG:HH12	2:B:571:ALA:HB2	1.71	0.55
3:C:134:THR:O	3:C:168:ASP:HB2	2.06	0.55
4:D:491:ILE:HG13	4:D:500:MSE:HE3	1.89	0.55
1:A:145:LEU:HD12	1:A:330:LYS:CD	2.36	0.55
1:A:150:MSE:HB3	1:A:158:VAL:HG21	1.88	0.55
3:C:681:LYS:NZ	3:C:682:LYS:O	2.30	0.55
1:A:71:VAL:HG23	1:A:101:ALA:HB2	1.89	0.55
3:C:656:MSE:HE1	3:C:672:SER:HB2	1.89	0.55
4:D:181:ARG:CZ	4:D:181:ARG:CB	2.83	0.55
4:D:706:ALA:HB3	4:D:731:SER:HB3	1.87	0.55
4:D:361:MSE:HE2	4:D:363:HIS:O	2.07	0.55
4:D:719:SER:OG	4:D:721:LYS:HG2	2.08	0.55
3:C:30:SER:HB3	3:C:95:TYR:CE1	2.42	0.54
2:B:431:HIS:HA	2:B:434:LYS:HE3	1.89	0.54
2:B:285:HIS:HE1	2:B:717:ASP:OD1	1.90	0.54
3:C:217:THR:OG1	3:C:220:SER:O	2.24	0.54
4:D:181:ARG:HG3	4:D:199:ALA:CB	2.38	0.54
2:B:181:ARG:N	2:B:181:ARG:HD3	2.23	0.54
2:B:238:MSE:HG2	2:B:239:LEU:N	2.21	0.54
3:C:544:HIS:HD2	3:C:546:GLY:H	1.54	0.54
2:B:305:ASP:HB2	2:B:307:THR:HG23	1.91	0.53
2:B:605:LEU:HD21	2:B:618:LYS:HG3	1.89	0.53
3:C:204:PHE:O	3:C:207:THR:OG1	2.22	0.53
2:B:318:LYS:HG2	2:B:400:VAL:HG21	1.91	0.53
3:C:169:ILE:HG13	3:C:193:VAL:HB	1.90	0.53
3:C:528:LEU:HD22	3:C:529:LEU:N	2.23	0.53
4:D:46:VAL:HG23	4:D:47:SER:N	2.22	0.53
1:A:197:ALA:HB3	1:A:203:LEU:HG	1.91	0.53
1:A:273:ASN:ND2	1:A:346:LEU:H	2.05	0.53
4:D:238:MSE:HE1	4:D:536:TRP:CE2	2.43	0.53
1:A:708:LEU:HD11	1:A:710:ASN:HB2	1.91	0.53
2:B:57:ASP:HB3	2:B:523:SER:HB3	1.91	0.53
2:B:113:ILE:HG13	2:B:209:THR:HG23	1.90	0.53
2:B:147:ALA:CB	2:B:160:LEU:HD21	2.39	0.53
1:A:22:ALA:C	1:A:24:GLN:H	2.12	0.53

Continued on next page...

PRELIMINARY VALIDATION REPORT

Continued from previous page...

Atom-1	Atom-2	Interatomic distance (Å)	Clash overlap (Å)
4:D:681:LYS:HD3	4:D:682:LYS:H	1.74	0.53
1:A:471:ALA:N	1:A:519:GLU:OE1	2.35	0.52
2:B:200:PRO:HA	2:B:203:LEU:HD12	1.91	0.52
3:C:580:HIS:O	3:C:584:VAL:HG23	2.09	0.52
1:A:499:ARG:HB3	1:A:502:ARG:HD2	1.90	0.52
3:C:113:ILE:HD11	3:C:213:MSE:CG	2.40	0.52
3:C:721:LYS:HA	3:C:723:LYS:HE2	1.92	0.52
4:D:578:GLU:N	4:D:578:GLU:OE1	2.43	0.52
2:B:447:ALA:O	2:B:448:LYS:HD3	2.09	0.52
4:D:386:PHE:CD1	4:D:390:MSE:HE3	2.45	0.52
3:C:232:TYR:CE2	3:C:513:PRO:HG2	2.45	0.52
3:C:706:ALA:HB3	3:C:731:SER:HB2	1.92	0.52
4:D:630:GLU:OE2	4:D:682:LYS:HE3	2.09	0.52
2:B:412:ASP:O	2:B:413:GLU:HB3	2.09	0.52
4:D:26:ALA:HA	4:D:29:LEU:HD13	1.91	0.52
1:A:145:LEU:HD12	1:A:330:LYS:HD2	1.92	0.51
3:C:342:PRO:HB2	3:C:370:MSE:HE3	1.92	0.51
4:D:238:MSE:HG3	4:D:267:HIS:CG	2.45	0.51
4:D:604:PRO:HG3	4:D:754:GLN:HE22	1.75	0.51
1:A:556:ILE:HG22	1:A:560:MSE:HE3	1.93	0.51
4:D:609:PRO:HG2	4:D:748:VAL:O	2.10	0.51
2:B:278:GLU:OE1	2:B:278:GLU:N	2.32	0.51
1:A:370:MSE:HE3	1:A:415:TYR:CE2	2.44	0.51
1:A:412:ASP:OD1	1:A:413:GLU:N	2.44	0.51
3:C:325:VAL:O	3:C:329:ARG:HG2	2.11	0.51
2:B:434:LYS:NZ	4:D:286:ASP:OD2	2.40	0.51
3:C:641:GLU:OE2	3:C:664:ARG:NH2	2.43	0.51
1:A:693:LYS:HE2	6:F:112:HOH:O	2.10	0.51
2:B:501:ASP:N	2:B:501:ASP:OD1	2.42	0.51
3:C:326:SER:HA	3:C:329:ARG:HG2	1.92	0.51
4:D:46:VAL:CG2	4:D:49:ALA:HB3	2.41	0.51
4:D:48:GLY:H	4:D:85:LYS:HZ1	1.57	0.51
1:A:392:LYS:HG3	3:C:364:GLU:OE2	2.11	0.50
2:B:614:GLN:OE1	2:B:616:LEU:HD23	2.11	0.50
3:C:171:LEU:HD22	3:C:195:LEU:HB3	1.91	0.50
3:C:486:ASP:N	3:C:486:ASP:OD1	2.44	0.50
3:C:656:MSE:HE1	3:C:672:SER:CB	2.41	0.50
4:D:656:MSE:HE1	4:D:672:SER:HB2	1.92	0.50
4:D:693:LYS:HA	4:D:731:SER:HB2	1.92	0.50
1:A:469:MSE:HE2	1:A:474:ALA:HA	1.92	0.50
1:A:670:GLU:N	1:A:710:ASN:OD1	2.38	0.50

Continued on next page...

PRELIMINARY VALIDATION REPORT

Continued from previous page...

Atom-1	Atom-2	Interatomic distance (Å)	Clash overlap (Å)
3:C:57:ASP:OD2	3:C:78:ASN:HB2	2.12	0.50
4:D:184:TYR:CE1	4:D:228:ASP:HB3	2.47	0.50
2:B:625:TYR:CZ	2:B:689:GLY:HA3	2.46	0.50
4:D:110:LYS:NZ	4:D:116:GLU:OE2	2.43	0.50
3:C:747:LYS:HD2	3:C:749:PHE:CZ	2.47	0.50
4:D:751:MSE:HE3	4:D:753:VAL:HG23	1.94	0.50
2:B:505:LYS:HG3	2:B:577:PHE:CE2	2.46	0.50
3:C:71:VAL:HG12	3:C:101:ALA:HB2	1.94	0.50
2:B:44:PRO:HG2	2:B:51:ILE:HG21	1.94	0.50
3:C:325:VAL:HA	3:C:335:ILE:HD12	1.92	0.50
3:C:301:MSE:HE3	3:C:385:VAL:HG22	1.94	0.49
4:D:309:LEU:HB2	4:D:351:LEU:HD11	1.93	0.49
4:D:556:ILE:O	4:D:560:MSE:HG3	2.12	0.49
1:A:230:PRO:HB3	1:A:568:TRP:CE3	2.47	0.49
4:D:205:TRP:CZ3	4:D:262:LYS:HA	2.47	0.49
3:C:93:LYS:HD3	3:C:93:LYS:N	2.28	0.49
1:A:238:MSE:HE2	1:A:534:ALA:HB1	1.94	0.49
1:A:556:ILE:CG2	1:A:560:MSE:HE3	2.43	0.49
2:B:273:ASN:HD21	2:B:369:GLU:CB	2.25	0.49
4:D:127:TYR:HB2	4:D:189:ALA:O	2.13	0.49
2:B:370:MSE:HE3	2:B:415:TYR:H	1.77	0.49
3:C:386:PHE:CD1	3:C:390:MSE:HE3	2.47	0.49
4:D:273:ASN:HD22	4:D:346:LEU:H	1.61	0.49
1:A:318:LYS:HG2	1:A:400:VAL:HG21	1.94	0.49
3:C:212:GLN:NE2	3:C:260:TRP:O	2.46	0.49
4:D:656:MSE:HE1	4:D:672:SER:CB	2.42	0.49
1:A:32:PRO:HB2	1:A:42:PRO:HB2	1.95	0.48
4:D:373:ALA:HB1	4:D:424:PHE:HB2	1.94	0.48
4:D:601:ASP:OD1	4:D:601:ASP:N	2.40	0.48
3:C:629:MSE:N	3:C:685:VAL:O	2.44	0.48
4:D:146:PHE:O	4:D:150:MSE:HG2	2.12	0.48
2:B:35:LYS:HG2	2:B:42:PRO:HG3	1.95	0.48
3:C:98:MSE:O	3:C:98:MSE:HG3	2.13	0.48
2:B:358:LYS:HA	2:B:367:ARG:HH11	1.79	0.48
3:C:110:LYS:HD2	3:C:117:ILE:HB	1.96	0.48
4:D:696:LEU:HD23	4:D:703:ALA:HB3	1.95	0.48
2:B:641:GLU:CD	2:B:664:ARG:HH22	2.16	0.48
4:D:370:MSE:HE3	4:D:415:TYR:CD2	2.48	0.48
4:D:661:VAL:HG12	4:D:673:PHE:HD2	1.79	0.48
2:B:370:MSE:HE3	2:B:415:TYR:N	2.29	0.48
4:D:48:GLY:H	4:D:85:LYS:NZ	2.11	0.48

Continued on next page...

PRELIMINARY VALIDATION REPORT

Continued from previous page...

Atom-1	Atom-2	Interatomic distance (Å)	Clash overlap (Å)
4:D:473:GLU:HG2	6:F:17:HOH:O	2.13	0.48
2:B:489:LEU:HB3	2:B:533:PHE:HA	1.95	0.48
2:B:655:VAL:HA	2:B:660:THR:O	2.13	0.48
4:D:63:ASN:ND2	4:D:67:LYS:HE3	2.28	0.48
3:C:74:ASP:HB3	3:C:98:MSE:HE1	1.96	0.48
3:C:486:ASP:HB2	3:C:498:TYR:CE1	2.49	0.48
1:A:627:LEU:O	1:A:686:GLU:HA	2.14	0.48
3:C:40:GLN:OE1	3:C:67:LYS:HD3	2.14	0.48
3:C:188:ILE:HG21	3:C:221:VAL:HG23	1.96	0.48
2:B:629:MSE:HE3	2:B:751:MSE:SE	2.64	0.47
1:A:417:ASP:OD2	1:A:418:LYS:N	2.46	0.47
2:B:312:ARG:C	2:B:312:ARG:HD2	2.34	0.47
3:C:238:MSE:HG3	3:C:267:HIS:CG	2.49	0.47
3:C:480:ASP:HB3	3:C:529:LEU:HD11	1.95	0.47
1:A:491:ILE:HG13	1:A:500:MSE:HE3	1.95	0.47
2:B:601:ASP:HB3	2:B:603:GLN:OE1	2.13	0.47
2:B:624:ASN:HA	2:B:689:GLY:O	2.15	0.47
3:C:31:ILE:CB	3:C:32:PRO:HD3	2.42	0.47
3:C:217:THR:OG1	3:C:217:THR:O	2.32	0.47
1:A:159:LYS:HE3	2:B:613:PRO:HD2	1.96	0.47
2:B:300:LYS:HG2	2:B:388:GLU:OE2	2.13	0.47
2:B:722:PHE:CE1	2:B:724:HIS:HB2	2.48	0.47
3:C:31:ILE:HG13	3:C:44:PRO:HB3	1.97	0.47
3:C:188:ILE:HG22	3:C:223:CYS:HA	1.95	0.47
2:B:112:ARG:O	2:B:113:ILE:HD13	2.14	0.47
2:B:154:LEU:HD21	2:B:214:LEU:HD13	1.96	0.47
2:B:159:LYS:NZ	2:B:161:VAL:HG12	2.29	0.47
2:B:440:ARG:HA	2:B:460:GLN:O	2.14	0.47
3:C:215:ARG:NH1	3:C:589:ASN:O	2.34	0.47
1:A:151:GLU:HG2	1:A:158:VAL:HG23	1.96	0.47
3:C:131:ASN:HB2	3:C:156:LYS:HG2	1.97	0.47
3:C:608:LYS:HE2	3:C:750:HIS:ND1	2.30	0.47
4:D:43:MSE:HE3	4:D:52:LYS:HD2	1.97	0.47
1:A:338:GLU:HG3	1:A:408:HIS:ND1	2.29	0.47
1:A:696:LEU:HG	1:A:703:ALA:HB3	1.96	0.47
3:C:146:PHE:CZ	3:C:150:MSE:HE3	2.50	0.47
1:A:271:ASN:HB3	1:A:295:PHE:HD1	1.80	0.46
2:B:205:TRP:CZ3	2:B:262:LYS:HA	2.49	0.46
2:B:600:LYS:HD2	2:B:600:LYS:N	2.29	0.46
1:A:469:MSE:HE2	1:A:474:ALA:CA	2.46	0.46
2:B:112:ARG:CZ	2:B:583:LEU:HD13	2.46	0.46

Continued on next page...

PRELIMINARY VALIDATION REPORT

Continued from previous page...

Atom-1	Atom-2	Interatomic distance (Å)	Clash overlap (Å)
2:B:212:GLN:HG2	2:B:260:TRP:CZ2	2.50	0.46
2:B:642:GLN:HB2	2:B:655:VAL:CG1	2.44	0.46
3:C:254:VAL:HG13	3:C:560:MSE:HE1	1.98	0.46
3:C:258:MSE:HG3	3:C:560:MSE:SE	2.65	0.46
3:C:447:ALA:HB2	3:C:468:TRP:HA	1.97	0.46
4:D:134:THR:HG22	4:D:159:LYS:HB3	1.96	0.46
1:A:63:ASN:OD1	1:A:67:LYS:N	2.42	0.46
3:C:156:LYS:HA	3:C:156:LYS:HD3	1.59	0.46
3:C:267:HIS:HE2	3:C:338:GLU:HB2	1.80	0.46
4:D:581:ARG:HA	4:D:581:ARG:HD3	1.75	0.46
2:B:433:LEU:HD22	4:D:354:ASP:HA	1.97	0.46
3:C:599:TRP:CD2	3:C:621:LEU:HD13	2.50	0.46
1:A:556:ILE:O	1:A:560:MSE:HG3	2.16	0.46
3:C:543:MSE:HG2	3:C:724:HIS:O	2.15	0.46
4:D:629:MSE:HE3	4:D:751:MSE:SE	2.65	0.46
2:B:697:LEU:HD23	2:B:702:PRO:CA	2.43	0.46
3:C:179:LEU:HD21	3:C:185:ARG:HG3	1.97	0.46
4:D:363:HIS:HD2	6:F:103:HOH:O	1.98	0.46
2:B:173:LEU:HD23	2:B:173:LEU:HA	1.82	0.46
4:D:473:GLU:O	4:D:477:GLN:HG3	2.16	0.46
1:A:26:ALA:HB1	1:A:92:SER:HA	1.98	0.45
4:D:235:ARG:HG2	4:D:567:LEU:HB3	1.98	0.45
1:A:436:GLY:C	3:C:358:LYS:HD3	2.37	0.45
2:B:446:SER:OG	2:B:473:GLU:OE1	2.26	0.45
3:C:601:ASP:OD2	3:C:603:GLN:HB2	2.16	0.45
1:A:250:TYR:O	1:A:254:VAL:HG23	2.15	0.45
3:C:171:LEU:HA	3:C:195:LEU:O	2.17	0.45
3:C:686:GLU:CG	3:C:697:LEU:HB2	2.44	0.45
2:B:693:LYS:C	2:B:693:LYS:HD3	2.37	0.45
3:C:390:MSE:HG2	3:C:437:TYR:CE2	2.51	0.45
4:D:661:VAL:HG12	4:D:673:PHE:CD2	2.51	0.45
1:A:271:ASN:HB3	1:A:295:PHE:CD1	2.51	0.45
2:B:656:MSE:HE1	2:B:672:SER:HB2	1.99	0.45
3:C:54:LEU:H	3:C:81:PHE:HA	1.81	0.45
3:C:203:LEU:HD23	3:C:203:LEU:HA	1.84	0.45
3:C:230:PRO:HD3	3:C:568:TRP:CH2	2.52	0.45
3:C:616:LEU:HD13	3:C:751:MSE:HE1	1.98	0.45
4:D:238:MSE:HE1	4:D:536:TRP:CD2	2.51	0.45
1:A:26:ALA:O	1:A:95:TYR:OH	2.23	0.45
3:C:126:GLU:HB2	3:C:220:SER:OG	2.17	0.45
3:C:370:MSE:HE2	3:C:414:PHE:CD1	2.48	0.45

Continued on next page...

PRELIMINARY VALIDATION REPORT

Continued from previous page...

Atom-1	Atom-2	Interatomic distance (Å)	Clash overlap (Å)
3:C:597:HIS:ND1	3:C:734:ILE:HD13	2.32	0.45
4:D:486:ASP:HB2	4:D:498:TYR:CE1	2.52	0.45
1:A:274:TYR:OH	1:A:543:MSE:HE2	2.17	0.45
1:A:543:MSE:HG2	1:A:724:HIS:O	2.17	0.45
2:B:664:ARG:HB2	2:B:670:GLU:HG2	1.99	0.45
2:B:722:PHE:HD2	2:B:725:ARG:HG2	1.78	0.45
2:B:40:GLN:O	2:B:68:ILE:HD12	2.17	0.45
1:A:189:ALA:HB3	1:A:191:ASP:OD1	2.17	0.45
1:A:489:LEU:HB3	1:A:533:PHE:HA	1.99	0.45
1:A:645:LEU:HB2	1:A:652:LEU:HB3	1.99	0.45
2:B:43:MSE:HE1	2:B:53:LEU:HB2	1.98	0.45
2:B:693:LYS:HE2	2:B:695:SER:HB3	1.99	0.45
3:C:267:HIS:NE2	3:C:338:GLU:OE1	2.51	0.44
3:C:408:HIS:HE1	3:C:442:TRP:CD1	2.35	0.44
4:D:82:LYS:HD3	4:D:89:GLU:OE2	2.17	0.44
4:D:641:GLU:CD	4:D:664:ARG:HH22	2.20	0.44
1:A:260:TRP:CE3	1:A:591:PRO:HD3	2.52	0.44
2:B:665:ARG:NH2	2:B:667:ASP:OD2	2.45	0.44
4:D:580:HIS:O	4:D:584:VAL:HG23	2.17	0.44
1:A:429:LEU:HD21	1:A:441:ILE:HB	1.99	0.44
2:B:81:PHE:HE2	2:B:95:TYR:HD2	1.65	0.44
1:A:297:LEU:HD23	1:A:297:LEU:HA	1.75	0.44
1:A:413:GLU:OE2	1:A:414:PHE:N	2.50	0.44
3:C:238:MSE:HE2	3:C:534:ALA:HB1	1.99	0.44
3:C:272:ASN:OD1	3:C:273:ASN:N	2.47	0.44
4:D:370:MSE:HE3	4:D:415:TYR:CG	2.52	0.44
2:B:627:LEU:O	2:B:686:GLU:HA	2.18	0.44
2:B:716:LYS:HB2	2:B:716:LYS:HE2	1.70	0.44
4:D:277:HIS:HE1	4:D:369:GLU:HB3	1.82	0.44
3:C:110:LYS:HA	3:C:120:TRP:CD1	2.52	0.44
3:C:293:ALA:HB2	3:C:346:LEU:HD23	2.00	0.44
1:A:118:LEU:N	1:A:228:ASP:OD1	2.40	0.44
1:A:613:PRO:HD3	2:B:159:LYS:HD2	1.99	0.44
1:A:186:LEU:HB3	1:A:226:ALA:HB3	2.00	0.44
1:A:370:MSE:HE3	1:A:415:TYR:CZ	2.53	0.44
3:C:473:GLU:O	3:C:477:GLN:HG3	2.18	0.44
1:A:291:SER:O	1:A:350:ARG:NH2	2.48	0.43
1:A:576:THR:HG23	1:A:579:GLN:NE2	2.33	0.43
1:A:608:LYS:HD3	1:A:749:PHE:O	2.17	0.43
2:B:338:GLU:OE2	2:B:340:ASP:OD1	2.36	0.43
4:D:471:ALA:N	4:D:519:GLU:OE1	2.35	0.43

Continued on next page...

PRELIMINARY VALIDATION REPORT

Continued from previous page...

Atom-1	Atom-2	Interatomic distance (Å)	Clash overlap (Å)
1:A:629:MSE:HE2	1:A:631:LEU:HD23	2.00	0.43
3:C:105:ALA:O	3:C:107:GLY:N	2.51	0.43
4:D:344:HIS:NE2	4:D:412:ASP:OD2	2.45	0.43
1:A:581:ARG:HD3	1:A:581:ARG:HA	1.78	0.43
2:B:713:ASP:O	2:B:716:LYS:NZ	2.51	0.43
3:C:210:LEU:HD23	3:C:213:MSE:CE	2.49	0.43
3:C:235:ARG:HG2	3:C:567:LEU:HB3	2.01	0.43
4:D:181:ARG:CZ	4:D:181:ARG:HB3	2.48	0.43
2:B:57:ASP:HB2	2:B:231:ARG:HD2	2.00	0.43
2:B:629:MSE:HG2	2:B:751:MSE:HG3	1.99	0.43
1:A:598:LYS:HA	1:A:598:LYS:HD2	1.80	0.43
1:A:609:PRO:HG2	1:A:748:VAL:HG12	1.99	0.43
3:C:79:VAL:O	3:C:95:TYR:N	2.51	0.43
3:C:156:LYS:HD3	3:C:157:LYS:N	2.33	0.43
4:D:262:LYS:HD3	4:D:568:TRP:CH2	2.53	0.43
4:D:751:MSE:HE3	4:D:753:VAL:CG2	2.48	0.43
2:B:50:GLN:O	2:B:83:VAL:HA	2.19	0.43
4:D:681:LYS:HD3	4:D:682:LYS:N	2.34	0.43
1:A:273:ASN:HD22	1:A:346:LEU:CB	2.32	0.43
2:B:297:LEU:HD23	2:B:297:LEU:HA	1.86	0.43
2:B:598:LYS:NZ	2:B:623:PRO:O	2.52	0.43
4:D:110:LYS:HD3	4:D:120:TRP:HB3	2.01	0.43
1:A:576:THR:OG1	1:A:579:GLN:HG3	2.19	0.43
2:B:61:LEU:HA	2:B:69:ALA:HB3	2.01	0.43
2:B:273:ASN:HD22	2:B:346:LEU:HB2	1.83	0.43
2:B:499:ARG:HB3	2:B:502:ARG:HD2	2.00	0.43
3:C:649:GLU:OE1	3:C:649:GLU:N	2.52	0.43
4:D:686:GLU:HB2	4:D:697:LEU:HB2	2.00	0.43
3:C:114:ILE:HD13	3:C:580:HIS:CE1	2.54	0.43
3:C:290:GLU:O	3:C:714:LYS:NZ	2.41	0.43
1:A:124:GLN:H	1:A:124:GLN:HG3	1.58	0.42
1:A:321:PHE:O	1:A:325:VAL:HG23	2.19	0.42
3:C:110:LYS:HD3	3:C:120:TRP:HB2	2.01	0.42
3:C:171:LEU:CD2	3:C:195:LEU:HB3	2.49	0.42
3:C:379:ILE:HD12	3:C:379:ILE:HA	1.94	0.42
4:D:215:ARG:HD3	4:D:215:ARG:HA	1.88	0.42
1:A:665:ARG:HB2	1:A:667:ASP:OD1	2.19	0.42
2:B:623:PRO:HA	2:B:624:ASN:HA	1.72	0.42
3:C:581:ARG:HD3	3:C:581:ARG:HA	1.64	0.42
4:D:181:ARG:HB2	4:D:181:ARG:CZ	2.47	0.42
4:D:398:ARG:NH2	4:D:403:ASP:OD1	2.52	0.42

Continued on next page...

PRELIMINARY VALIDATION REPORT

Continued from previous page...

Atom-1	Atom-2	Interatomic distance (Å)	Clash overlap (Å)
3:C:475:VAL:HG11	3:C:525:HIS:NE2	2.34	0.42
3:C:601:ASP:CG	3:C:603:GLN:H	2.22	0.42
4:D:339:PHE:HB2	4:D:386:PHE:CZ	2.54	0.42
1:A:715:SER:O	1:A:718:PHE:HB2	2.20	0.42
2:B:475:VAL:HG11	2:B:525:HIS:NE2	2.34	0.42
3:C:665:ARG:HB2	3:C:667:ASP:OD1	2.20	0.42
1:A:734:ILE:O	1:A:736:PRO:HD3	2.19	0.42
3:C:257:THR:O	3:C:260:TRP:HB3	2.18	0.42
1:A:522:PRO:HG2	1:A:525:HIS:HB2	2.02	0.42
1:A:651:GLU:OE2	1:A:664:ARG:NH1	2.53	0.42
2:B:212:GLN:HG2	2:B:260:TRP:CH2	2.54	0.42
2:B:313:ASP:OD1	2:B:314:LEU:N	2.53	0.42
1:A:238:MSE:HG2	1:A:239:LEU:N	2.34	0.42
1:A:276:PHE:CE2	1:A:543:MSE:HE1	2.55	0.42
1:A:419:GLU:OE1	1:A:452:THR:OG1	2.26	0.42
2:B:200:PRO:O	2:B:203:LEU:HB2	2.20	0.42
2:B:638:GLU:OE1	2:B:638:GLU:N	2.48	0.42
2:B:722:PHE:C	2:B:724:HIS:H	2.23	0.42
3:C:85:LYS:HD2	3:C:85:LYS:HA	1.69	0.42
3:C:260:TRP:CE3	3:C:591:PRO:HD3	2.54	0.42
4:D:138:PRO:O	4:D:140:LYS:NZ	2.50	0.42
4:D:641:GLU:OE2	4:D:664:ARG:NH2	2.43	0.42
2:B:464:TRP:CE3	2:B:465:SER:HB2	2.55	0.42
3:C:146:PHE:CE2	3:C:211:LEU:HD21	2.55	0.42
3:C:171:LEU:HD13	3:C:203:LEU:HD22	2.00	0.42
3:C:561:ASP:OD1	3:C:562:VAL:N	2.52	0.42
4:D:181:ARG:HB2	4:D:181:ARG:HH21	1.84	0.42
4:D:489:LEU:HB3	4:D:533:PHE:HA	2.01	0.42
2:B:52:LYS:HG2	2:B:53:LEU:N	2.35	0.41
3:C:383[A]:SER:HB2	3:C:431:HIS:CE1	2.54	0.41
4:D:297:LEU:HD23	4:D:297:LEU:HA	1.87	0.41
2:B:645:LEU:HB2	2:B:652:LEU:HB3	2.02	0.41
3:C:184:TYR:CE1	3:C:228:ASP:HB3	2.55	0.41
4:D:135:ILE:HD11	4:D:150:MSE:HG3	2.02	0.41
1:A:431:HIS:O	1:A:434:LYS:HG2	2.20	0.41
2:B:145:LEU:CD2	2:B:330:LYS:HG2	2.50	0.41
2:B:709:LYS:HA	2:B:709:LYS:HD2	1.78	0.41
1:A:76:PRO:HB3	1:A:98:MSE:HE2	2.02	0.41
3:C:232:TYR:CE1	3:C:528:LEU:HD13	2.56	0.41
3:C:255:ILE:HD11	3:C:335:ILE:HD11	2.02	0.41
3:C:370:MSE:CE	3:C:414:PHE:HA	2.47	0.41

Continued on next page...

PRELIMINARY VALIDATION REPORT

Continued from previous page...

Atom-1	Atom-2	Interatomic distance (Å)	Clash overlap (Å)
4:D:664:ARG:HH11	4:D:670:GLU:CD	2.24	0.41
1:A:505:LYS:HG3	1:A:577:PHE:CD2	2.55	0.41
1:A:613:PRO:CD	2:B:159:LYS:HD2	2.51	0.41
2:B:115:PRO:HD2	2:B:262:LYS:HD2	2.02	0.41
2:B:348:PHE:CE1	2:B:385:VAL:HG21	2.54	0.41
3:C:139:ASP:HB3	3:C:142:LEU:HB3	2.01	0.41
1:A:599:TRP:CG	1:A:621:LEU:HD13	2.56	0.41
2:B:655:VAL:HG23	2:B:659:GLY:O	2.20	0.41
3:C:372:ASP:HB3	3:C:375:ASN:HB2	2.01	0.41
3:C:645:LEU:HB2	3:C:652:LEU:HB3	2.03	0.41
3:C:556:ILE:O	3:C:560:MSE:HG3	2.21	0.41
4:D:548:ALA:HB2	4:D:665:ARG:CZ	2.50	0.41
1:A:313:ASP:OD1	1:A:314:LEU:N	2.52	0.41
2:B:57:ASP:OD1	2:B:57:ASP:N	2.53	0.41
2:B:607:VAL:CG2	2:B:751:MSE:HB3	2.51	0.41
3:C:373:ALA:HB1	3:C:424:PHE:HB2	2.03	0.41
3:C:419:GLU:CD	3:C:451:LYS:H	2.24	0.41
3:C:546:GLY:HA2	3:C:708:LEU:HD22	2.03	0.41
4:D:213:MSE:HE3	4:D:222:PRO:HD2	2.03	0.41
2:B:238:MSE:CE	2:B:534:ALA:HB1	2.43	0.41
2:B:341:THR:HG21	2:B:382:VAL:HG22	2.02	0.41
3:C:209:THR:HG22	3:C:213:MSE:HE2	2.03	0.41
3:C:511:TRP:O	3:C:566:LYS:HE3	2.21	0.41
3:C:605:LEU:O	3:C:752:ASN:HA	2.21	0.41
4:D:355:LEU:HD13	4:D:378:THR:HA	2.03	0.41
2:B:112:ARG:C	2:B:113:ILE:HD13	2.41	0.40
1:A:623:PRO:HA	1:A:624:ASN:HA	1.80	0.40
1:A:654:ALA:O	1:A:661:VAL:HG22	2.21	0.40
2:B:317:THR:HG23	2:B:320:GLU:OE1	2.21	0.40
3:C:528:LEU:HD22	3:C:530:GLY:N	2.31	0.40
2:B:599:TRP:CD2	2:B:621:LEU:HD13	2.57	0.40
2:B:629:MSE:HE1	2:B:644:LEU:HD21	2.02	0.40
3:C:244:THR:OG1	3:C:544:HIS:HE1	2.05	0.40
4:D:238:MSE:HE2	4:D:534:ALA:HB1	2.03	0.40
4:D:238:MSE:HA	4:D:267:HIS:HB3	2.02	0.40
1:A:525:HIS:HA	1:A:526:PRO:HD3	1.93	0.40
1:A:114:ILE:HA	1:A:115:PRO:HA	1.82	0.40
1:A:271:ASN:HA	1:A:294:ALA:O	2.22	0.40
2:B:134:THR:OG1	2:B:167:ALA:HA	2.22	0.40
3:C:156:LYS:HD3	3:C:157:LYS:H	1.86	0.40
3:C:356:ILE:HD13	3:C:368:CYS:HA	2.03	0.40

Continued on next page...

PRELIMINARY VALIDATION REPORT

Continued from previous page...

Atom-1	Atom-2	Interatomic distance (Å)	Clash overlap (Å)
3:C:464:TRP:HA	3:C:484:THR:O	2.22	0.40
4:D:157:LYS:HA	4:D:157:LYS:HD3	1.86	0.40

All (1) symmetry-related close contacts are listed below. The label for Atom-2 includes the symmetry operator and encoded unit-cell translations to be applied.

Atom-1	Atom-2	Interatomic distance (Å)	Clash overlap (Å)
2:B:362:ASN:OD1	4:D:612:LEU:N[2_555]	2.16	0.04

5.3 Torsion angles (i)

5.3.1 Protein backbone (i)

In the following table, the Percentiles column shows the percent Ramachandran outliers of the chain as a percentile score with respect to all X-ray entries followed by that with respect to entries of similar resolution.

The Analysed column shows the number of residues for which the backbone conformation was analysed, and the total number of residues.

Mol	Chain	Analysed	Favoured	Allowed	Outliers	Percentiles	
1	A	737/737 (100%)	711 (96%)	20 (3%)	6 (1%)	19	47
2	B	728/727 (100%)	687 (94%)	35 (5%)	6 (1%)	19	47
3	C	733/731 (100%)	693 (94%)	35 (5%)	5 (1%)	22	51
4	D	737/734 (100%)	706 (96%)	29 (4%)	2 (0%)	41	70
All	All	2935/2929 (100%)	2797 (95%)	119 (4%)	19 (1%)	22	54

All (19) Ramachandran outliers are listed below:

Mol	Chain	Res	Type
1	A	23	LYS
2	B	31	ILE
3	C	27	ASP
3	C	46	VAL
3	C	168	ASP
1	A	33	PRO
1	A	34	VAL
1	A	717	ASP

Continued on next page...

PRELIMINARY VALIDATION REPORT

Continued from previous page...

Mol	Chain	Res	Type
3	C	31	ILE
3	C	106	GLN
2	B	140	LYS
4	D	46	VAL
1	A	713	ASP
2	B	33	PRO
2	B	106	GLN
2	B	47	SER
2	B	725	ARG
1	A	163	PRO
4	D	34	VAL

5.3.2 Protein sidechains (i)

In the following table, the Percentiles column shows the percent sidechain outliers of the chain as a percentile score with respect to all X-ray entries followed by that with respect to entries of similar resolution.

The Analysed column shows the number of residues for which the sidechain conformation was analysed, and the total number of residues.

Mol	Chain	Analysed	Rotameric	Outliers	Percentiles	
1	A	619/598 (104%)	618 (100%)	1 (0%)	93	98
2	B	612/590 (104%)	611 (100%)	1 (0%)	93	98
3	C	616/593 (104%)	615 (100%)	1 (0%)	93	98
4	D	620/596 (104%)	619 (100%)	1 (0%)	93	98
All	All	2467/2377 (104%)	2463 (100%)	4 (0%)	93	98

All (4) residues with a non-rotameric sidechain are listed below:

Mol	Chain	Res	Type
1	A	312	ARG
2	B	413	GLU
3	C	157	LYS
4	D	181	ARG

Sometimes sidechains can be flipped to improve hydrogen bonding and reduce clashes. All (12) such sidechains are listed below:

Mol	Chain	Res	Type
1	A	273	ASN

Continued on next page...

PRELIMINARY VALIDATION REPORT

Continued from previous page...

Mol	Chain	Res	Type
1	A	579	GLN
2	B	100	GLN
2	B	273	ASN
2	B	285	HIS
2	B	745	GLN
3	C	544	HIS
4	D	273	ASN
4	D	277	HIS
4	D	363	HIS
4	D	745	GLN
4	D	754	GLN

5.3.3 RNA (i)

There are no RNA molecules in this entry.

5.4 Non-standard residues in protein, DNA, RNA chains (i)

There are no non-standard protein/DNA/RNA residues in this entry.

5.5 Carbohydrates (i)

There are no monosaccharides in this entry.

5.6 Ligand geometry (i)

Of 4 ligands modelled in this entry, 4 are monoatomic - leaving 0 for Mogul analysis.

There are no bond length outliers.

There are no bond angle outliers.

There are no chirality outliers.

There are no torsion outliers.

There are no ring outliers.

No monomer is involved in short contacts.

5.7 Other polymers (i)

There are no such residues in this entry.

PRELIMINARY VALIDATION REPORT

5.8 Polymer linkage issues (i)

There are no chain breaks in this entry.

PRELIMINARY VALIDATION REPORT

6 Fit of model and data (i)

6.1 Protein, DNA and RNA chains (i)

In the following table, the column labelled '#RSRZ> 2' contains the number (and percentage) of RSRZ outliers, followed by percent RSRZ outliers for the chain as percentile scores relative to all X-ray entries and entries of similar resolution. The OWAB column contains the minimum, median, 95th percentile and maximum values of the occupancy-weighted average B-factor per residue. The column labelled 'Q< 0.9' lists the number of (and percentage) of residues with an average occupancy less than 0.9.

Mol	Chain	Analysed	<RSRZ>	#RSRZ>2			OWAB(Å^2)	Q<0.9
1	A	718/737 (97%)	-0.08	26 (3%)	42	32	30, 46, 78, 141	0
2	B	708/727 (97%)	0.01	35 (4%)	29	20	28, 50, 97, 157	0
3	C	712/731 (97%)	0.01	40 (5%)	24	16	26, 50, 97, 145	0
4	D	715/734 (97%)	-0.35	11 (1%)	73	67	21, 37, 69, 123	0
All	All	2853/2929 (97%)	-0.10	112 (3%)	39	29	21, 46, 89, 157	0

All (112) RSRZ outliers are listed below:

Mol	Chain	Res	Type	RSRZ
2	B	720	ASP	8.7
2	B	50	GLN	6.6
1	A	104	ALA	6.2
3	C	164	GLY	5.6
1	A	720	ASP	5.6
1	A	721	LYS	5.5
2	B	166	LYS	5.3
3	C	163	PRO	4.9
2	B	164	GLY	4.9
3	C	46	VAL	4.8
2	B	719	SER	4.8
2	B	87	GLY	4.6
2	B	721	LYS	4.5
1	A	719	SER	4.5
3	C	104	ALA	4.4
2	B	86	ASP	4.3
4	D	163	PRO	4.3
3	C	103	GLN	4.3
2	B	84	THR	4.2
3	C	86	ASP	4.1
2	B	85	LYS	4.1

Continued on next page...

PRELIMINARY VALIDATION REPORT

Continued from previous page...

Mol	Chain	Res	Type	RSRZ
3	C	106	GLN	4.1
3	C	105	ALA	4.1
3	C	84	THR	4.1
3	C	162	ALA	4.0
1	A	84	THR	3.9
2	B	106	GLN	3.9
3	C	176	GLY	3.8
1	A	105	ALA	3.8
3	C	394	PRO	3.8
3	C	166	LYS	3.7
3	C	85	LYS	3.5
3	C	49	ALA	3.5
1	A	608	LYS	3.5
1	A	106	GLN	3.4
2	B	32	PRO	3.3
2	B	176	GLY	3.2
2	B	163	PRO	3.2
4	D	164	GLY	3.1
2	B	601	ASP	3.1
1	A	50	GLN	3.1
3	C	124	GLN	3.1
3	C	100	GLN	3.0
3	C	87	GLY	2.9
2	B	610	SER	2.9
3	C	47	SER	2.9
4	D	87	GLY	2.9
3	C	102	PRO	2.9
3	C	45	SER	2.9
1	A	87	GLY	2.9
3	C	88	LYS	2.8
4	D	50	GLN	2.8
3	C	51	ILE	2.8
3	C	206	GLY	2.8
1	A	166	LYS	2.8
1	A	165	ALA	2.8
1	A	209	THR	2.8
4	D	86	ASP	2.8
2	B	46	VAL	2.8
2	B	162	ALA	2.7
3	C	50	GLN	2.7
2	B	88	LYS	2.7
4	D	601	ASP	2.7

Continued on next page...

PRELIMINARY VALIDATION REPORT

Continued from previous page...

Mol	Chain	Res	Type	RSRZ
2	B	638	GLU	2.7
1	A	47	SER	2.6
2	B	718	PHE	2.6
1	A	175	LYS	2.6
3	C	412	ASP	2.5
4	D	175	LYS	2.5
2	B	209	THR	2.5
3	C	161	VAL	2.5
1	A	582	GLU	2.4
4	D	141	GLU	2.4
1	A	164	GLY	2.4
3	C	37	GLY	2.4
3	C	31	ILE	2.4
2	B	89	GLU	2.4
1	A	86	ASP	2.4
3	C	175	LYS	2.4
3	C	130	GLY	2.4
2	B	194	ARG	2.4
3	C	83	VAL	2.3
3	C	138	PRO	2.3
3	C	207	THR	2.3
2	B	617	ASP	2.3
2	B	52	LYS	2.3
3	C	131	ASN	2.3
2	B	608	LYS	2.3
1	A	206	GLY	2.3
3	C	568	TRP	2.2
4	D	610	SER	2.2
1	A	722	PHE	2.2
4	D	88	LYS	2.2
3	C	395	LYS	2.2
1	A	35	LYS	2.2
1	A	49	ALA	2.2
2	B	175	LYS	2.2
2	B	49	ALA	2.2
3	C	190	ARG	2.2
2	B	630	GLU	2.2
3	C	33	PRO	2.1
2	B	47	SER	2.1
2	B	132	THR	2.1
1	A	124	GLN	2.1
1	A	107	GLY	2.0

Continued on next page...

PRELIMINARY VALIDATION REPORT

Continued from previous page...

Mol	Chain	Res	Type	RSRZ
1	A	207	THR	2.0
3	C	48	GLY	2.0
2	B	306	GLY	2.0
4	D	166	LYS	2.0
1	A	306	GLY	2.0
2	B	579	GLN	2.0
2	B	611	SER	2.0

6.2 Non-standard residues in protein, DNA, RNA chains (i)

There are no non-standard protein/DNA/RNA residues in this entry.

6.3 Carbohydrates (i)

There are no monosaccharides in this entry.

6.4 Ligands (i)

In the following table, the Atoms column lists the number of modelled atoms in the group and the number defined in the chemical component dictionary. The B-factors column lists the minimum, median, 95th percentile and maximum values of B factors of atoms in the group. The column labelled 'Q< 0.9' lists the number of atoms with occupancy less than 0.9.

Mol	Type	Chain	Res	Atoms	RSCC	RSR	B-factors(Å^2)	Q<0.9
5	MG	E	3	1/?	0.97	0.27	29,29,29,29	0
5	MG	E	2	1/?	0.98	0.26	23,23,23,23	0
5	MG	E	1	1/?	0.99	0.26	22,22,22,22	0
5	MG	E	4	1/?	0.99	0.30	24,24,24,24	0

6.5 Other polymers (i)

There are no such residues in this entry.

PRELIMINARY VALIDATION REPORT
