# Supplementary figures and images for: Nucleotide binding as an allosteric regulatory mechanism for Akkermansia muciniphila β-N-acetylhexosaminidase Am2136
Source: Gut Microbes. 2022 Nov 17;14(1):2143221. doi: 10.1080/19490976.2022.2143221 (PMC9673926; doi:10.1080/19490976.2022.2143221)

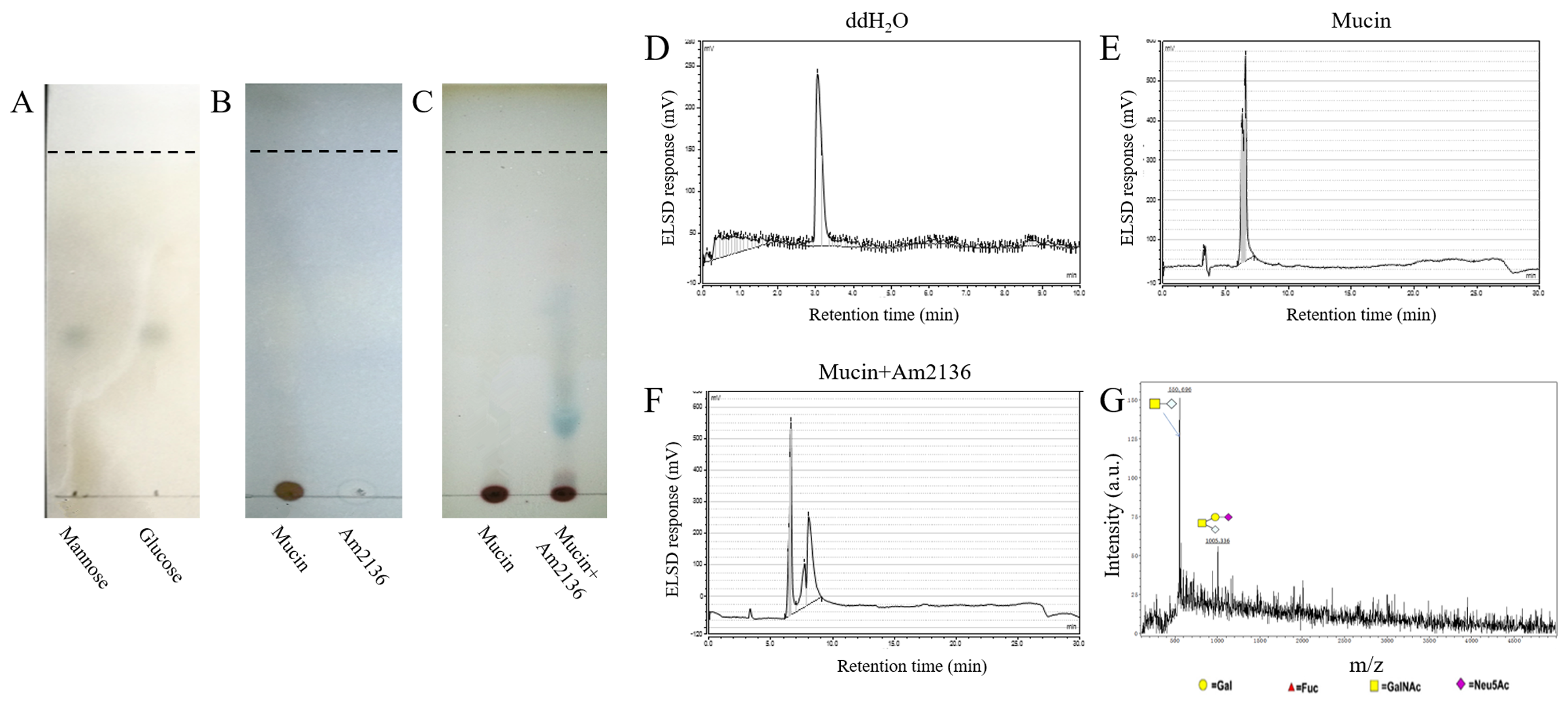

Supplement: Supplemental Material [file KGMI_A_2143221_SM4661.zip › Fig S1.tif]

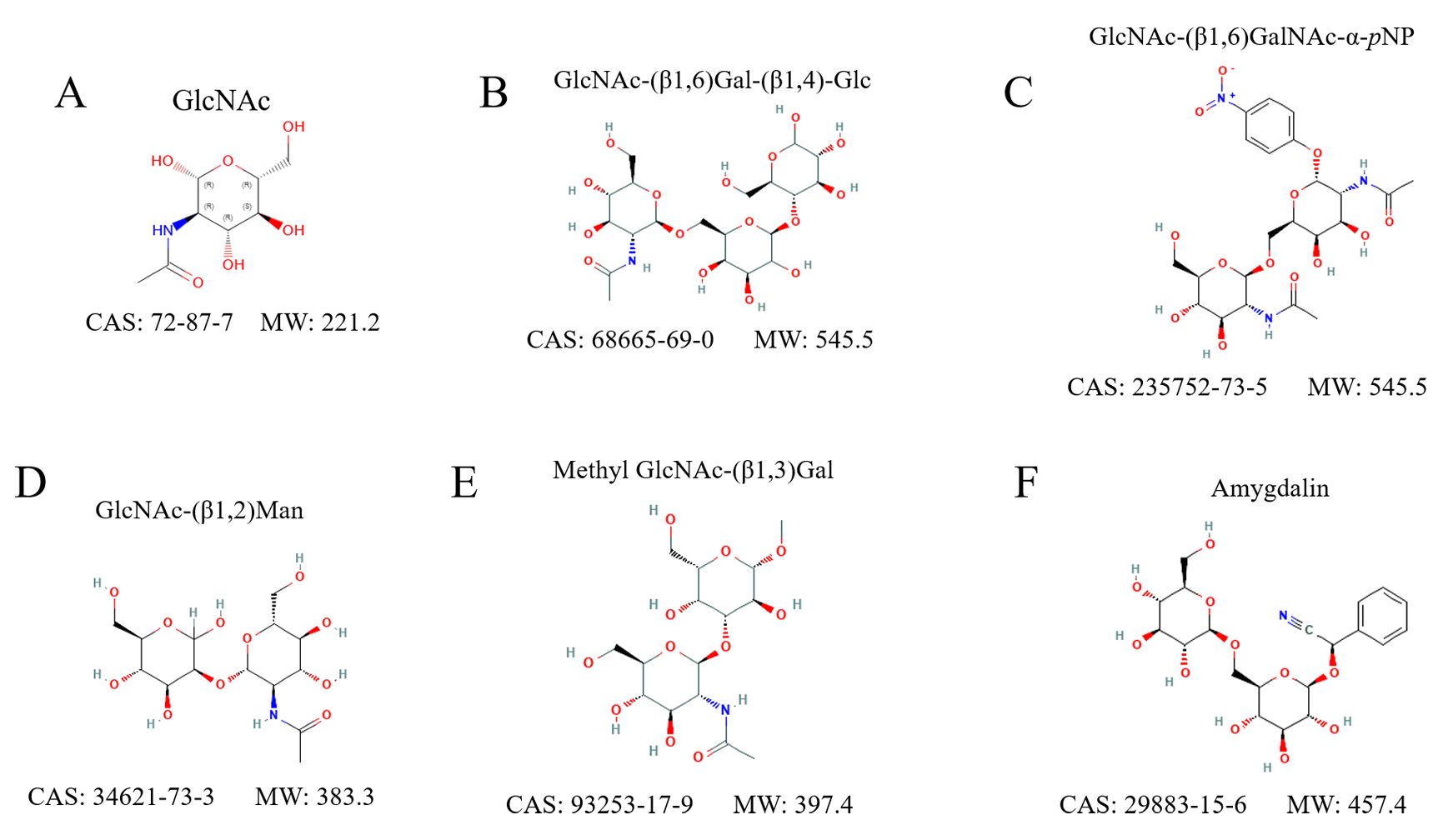

Supplement: Supplemental Material [file KGMI_A_2143221_SM4661.zip › Fig S2.tif]

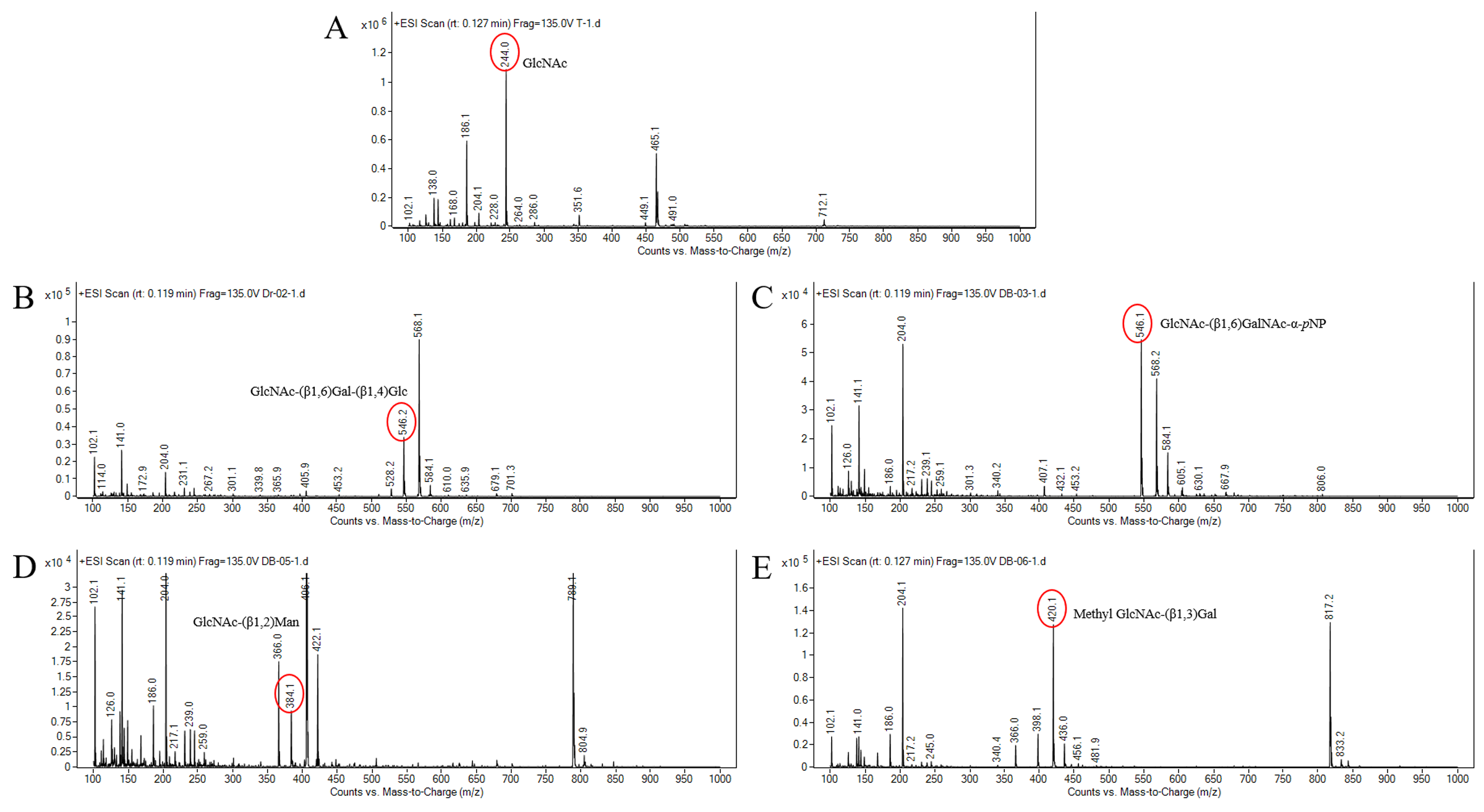

Supplement: Supplemental Material [file KGMI_A_2143221_SM4661.zip › Fig S3.tif]

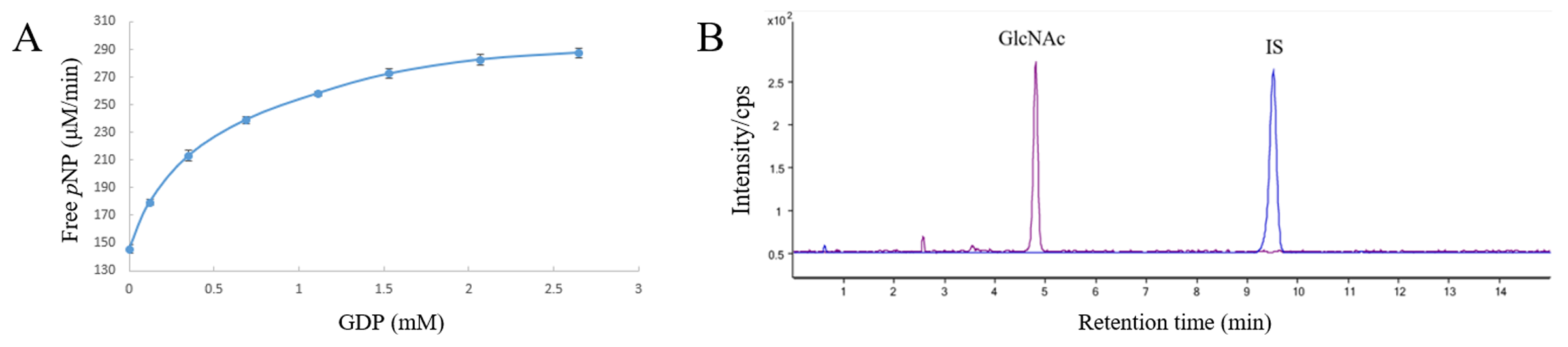

Supplement: Supplemental Material [file KGMI_A_2143221_SM4661.zip › Fig S4.tif]

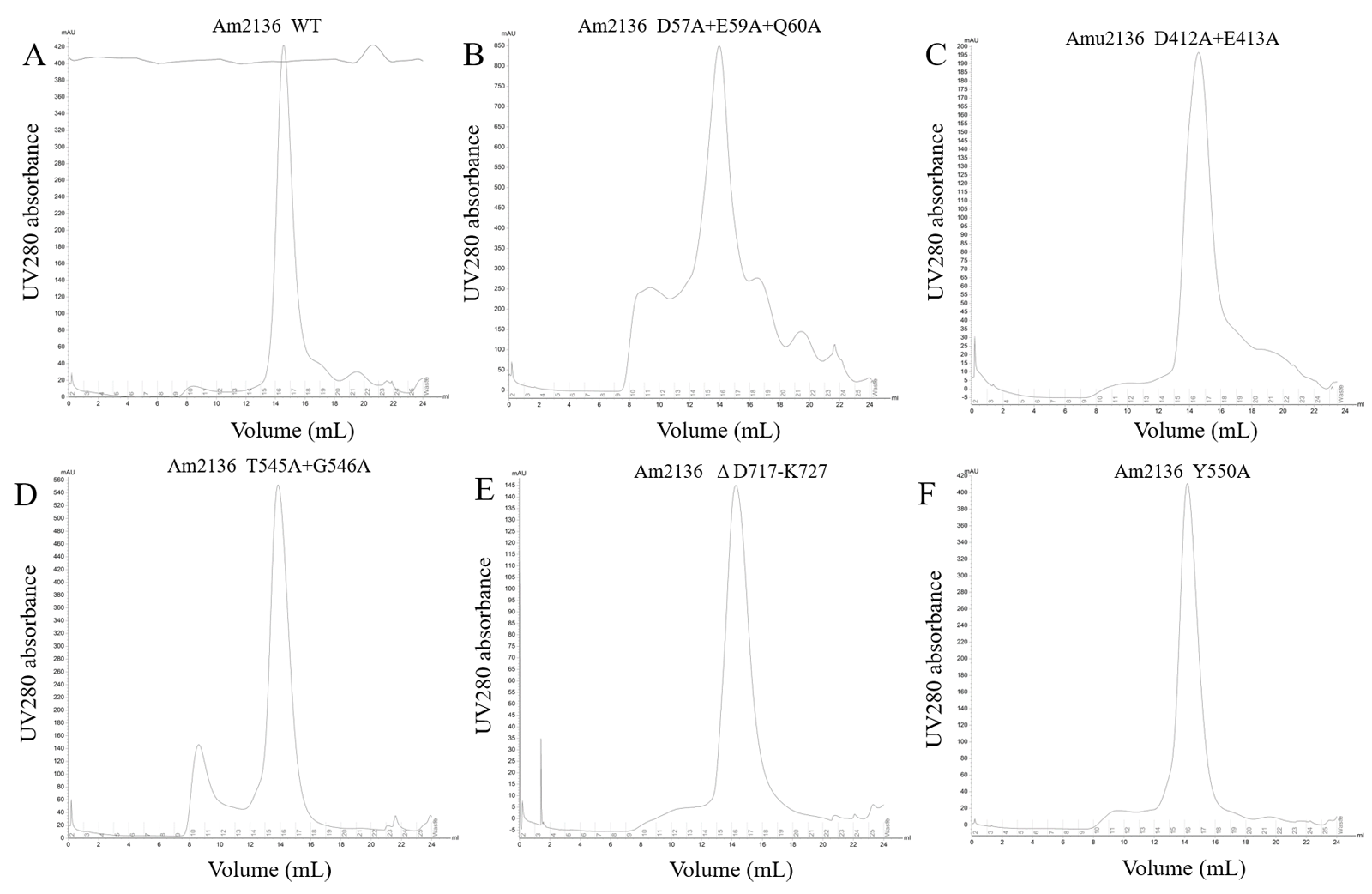

Supplement: Supplemental Material [file KGMI_A_2143221_SM4661.zip › Fig S5.tif]
